# Unraveling developmental gene regulation in holometabolous insects through comparative transcriptomics and proteomics

**DOI:** 10.1038/s42003-025-08414-z

**Published:** 2025-07-01

**Authors:** Maya Wilkens, Susanne Zimbelmann, Franziska Roth, Jasmin Cartano, Sergi Sayols, Mario Dejung, Michal Levin, Falk Butter

**Affiliations:** 1 Institute of Molecular Biology (IMB), Ackermannweg 4, Mainz, Germany; 2 Johannes Gutenberg University Mainz, Institute of Developmental Biology and Neurobiology (IDN), Mainz, Germany; 3 Friedrich-Loeffler-Institut (FLI), Südufer 10, Greifswald, Germany

**Keywords:** Data integration, Systems analysis

## Abstract

Holometabolous insects undergo complex phenotypic changes during development in four major stages: egg, larva, pupa and adult. Such changes are typically driven by strong transcriptome and proteome dynamics, making this process an excellent system for comparing these two levels of regulation. Here, we provide a comprehensive paired transcriptome and proteome dataset of 17 timepoints across the developmental life cycle of the silkworm *Bombyx mori*. The analysis of this data revealed similarities and differences between transcriptional and post-transcriptional gene expression, enabling the identification of stage-specific characteristics. Specifically, the oxidative phosphorylation pathway was enriched in genes expressed especially in adults. We examined protein-transcript correlations and characterized stage-specific dynamics. The majority of genes for which transcript and protein dynamics differ are linked to translation and RNA regulation. Our data constitute a rich resource enabling comparative analysis of developmental regulatory dynamics. Comparison of silkworm developmental gene expression with publicly available data for *D. melanogaster* revealed similar gene regulatory patterns at the transcriptome and proteome levels, underscoring the importance of the evolutionary conservation of tightly coordinated developmental processes.

## Abstract (web summary)

Paired transcriptome and proteome profiling throughout the silkworm’s developmental life cycle offers a comprehensive resource revealing stage-specific and conserved expression dynamics across holometabolous insects.

## Introduction

Metazoan development progresses through a series of cellular states, each defined by distinct changes in gene expression. Transcriptomic studies across developmental time courses have provided a deeper understanding of the intricate control of this process (reviewed in refs. ^1,2^). Studies on transcript expression during development have been conducted across multiple species, including *Drosophila melanogaster*^3,4^, *Caenorhabditis elegans*^5^, *Aedes aegypti*^6^, and *Daphnia mitsukuri*^7^, as well as multi-species studies encompassing different nematode species^8^, and a study involving ten metazoan species from different phyla^9^.

These studies often assume that mRNA dynamics reflect proteomic changes. However, transcript levels have been demonstrated to only moderately correlate with proteome levels in multiple species, as they do not account for post-transcriptional processes^10–15^. Hence, multiple studies have investigated proteomic changes across development by directly measuring and analyzing proteomes at different stages in *D. melanogaster*^12^, *Crassostrea gigas*^16^ and *Maruca vitrata*^17^.

Integrating transcriptome and proteome dynamics throughout developmental stages enables the most comprehensive understanding of the different levels of gene regulation and has been performed in different organisms, including *Xenopus laevis*^18,19^, *C. elegans*^15^, *Platynereis dumerilii*^11^, *Mus musculus*^20–22^, and *D. melanogaster*^23^. In addition to studying expression dynamics in individual species, comparative approaches across different species provide valuable insights into the evolutionary dynamics of developmental processes. Studies of different nematode species^8,15^, different frog species^24^, different insects^25^, and animals from different phyla^9,26^ have revealed striking similarities but also divergent aspects in their developmental regulation. Many of these studies concluded that the expression of orthologs in metazoans correlates remarkably well across species, while the correlation is not uniform across all stages, i.e., some stages exhibit greater similarity than others.

A common developmental strategy among insects is holometabolism, which is observed in 80% of this class^27^ and is characterized by complete metamorphosis through four stages (egg, larva, pupa, and adult). Transitions between these individual stages rely on tightly regulated gene expression mechanisms at the transcriptional, epigenetic and translational levels^28^. Holometabola comprises 11 orders^29^, including Diptera, which includes the well-studied model organism *D. melanogaster*, for which extensive developmental transcriptome and proteome studies have been conducted^4,12^. Another frequently used model insect is the lepidopteran species *Bombyx mori*, which is commonly known as the silkworm due to its economic importance in silk production. It is used as an animal model in various fields of research, such as human disease^30^, environmental monitoring^31^, toxicology^32^, epigenetics^33^, genetic engineering^34^, drug screening and discovery^35^, and evolutionary studies^36^. The aforementioned applications have established *B. mori* as one of the most frequently used model insects in modern research^37^. The two holometabolous species *D. melanogaster* and *B. mori*, which belong to the monophyletic clade Mecopterida^29^, share a last common ancestor (LCA) in the Late Carboniferous to Early Permian ~300 million years ago^38^, which is as far as the LCA estimated for mammals and reptiles^39^. Based on the overall similarities in their developmental life cycles, these two insect species are highly interesting for comparative systems biology. For *B. mori*, transcriptome data covering the full life cycle is available^40^. However, matching proteome data is lacking, precluding analysis at both gene regulatory levels. To account for this, we provide the developmental proteome of the complete life cycle of *B. mori* encompassing expression dynamics of 6058 proteins at 17 timepoints. To enable direct comparison of proteomic and transcriptomic changes and exclude biological variations, we also measured the transcriptomes of the same samples using next-generation RNA sequencing. By analyzing this paired dataset, we not only revealed global trends at both levels but also investigated protein-transcript correlations and characterized their stage-specific dynamics. Furthermore, we compared our data with the developmental transcriptome^4^ and proteome^12^ of *D. melanogaster* to provide a valuable perspective on the conserved and divergent aspects of developmental regulation across these two holometabolous insects.

## Results

### *Bombyx mori* developmental proteome shows the importance of OXPHOS in adults

**Fig. 1:**
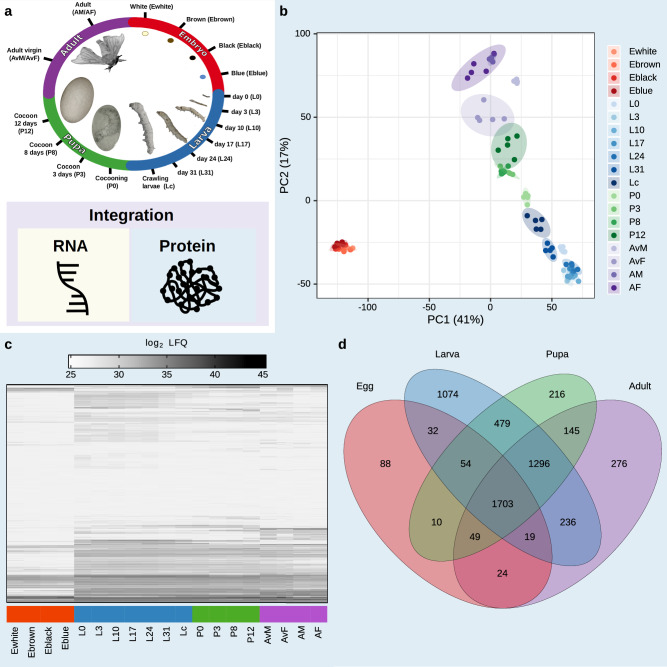
Developmental proteome of *Bombyx mori.* **a** Overview of the life cycle and timepoints sampled throughout the four major metamorphic developmental stages (egg (red), larva (blue), pupa (green), and adult (purple)). The lower panel depicts the color code for the data types used throughout the manuscript (RNA (yellow), protein (blue), and RNA–protein comparative analyses (purple)). **b** Scatter plot of the first two principal components of all measured samples. Biological replicates are shown in the same color, with elliptical areas representing the standard deviation between replicates. **c** Heatmap of average protein intensities (mean log_2_ LFQ intensities, *n* = 5 biological replicates) of the 6058 quantified proteins across all timepoints. **d** Venn diagram depicting overlaps of proteins between developmental stages with a core proteome of 1703 proteins quantified across all stages.

To determine the developmental proteome of *B. mori*, we collected whole-animal samples at 17 different timepoints throughout its life cycle. These timepoints cover the four major developmental stages: egg (4), larva (8), pupa (3) and adult (2 per sex, Fig. 1a). The egg timepoints were distinguished by color: freshly laid eggs (Ewhite), a few hours old (Ebrown), several days old (Eblack) and just before hatching (Eblue). For larval timepoints, specimens were harvested directly after hatching (L0) and on days 3, 10, 17, 24, and 31 post-hatching (L3, L10, L17, L24, and L31, respectively). In addition, wandering larvae (Lc) and pupae without their cocoon were collected on days 0, 3, 8, and 12 post pupation (P0, P3, P8 and P12, respectively). Adults included both virgin females and virgin males (AvF and AvM) and at 5 days post mating after egg laying (AF and AM). Quintuplicates for each timepoint were used to measure both transcriptome and proteome from the same sample (Supplementary Fig. S1). For proteome quantification, these 95 samples (5 replicates per timepoint) were measured by single-shot proteomics on a high-resolution mass spectrometer. Each replicate was measured with a 2.5 h gradient, amounting to a total measuring time of 238 h. The resulting spectra were searched against a combined protein database of *B. mori* and *Morus notabilis*, the larval mulberry food source. Overall, 6157 proteins were quantified by MaxLFQ^41^, including 6058 silkworm and 99 mulberry proteins (Supplementary Fig. S2). Replicates of each timepoint exhibited high reproducibility with high Pearson correlation coefficients (Pearson’s *R* = 0.93–0.98, Supplementary Fig. S3). Visualizing the first two PCA components, replicates clustered closely together, while individual timepoints revealed a developmental trajectory from the egg through larval and pupal stages to the adult stage, with eggs being the most divergent (Fig. 1b). This is also apparent in the expression profiles of the individual proteins (Fig. 1c). We compared the detection of proteins in the four main life cycle stages to provide a global assessment of the developmental proteome. The majority of stage-specific proteins were quantified in the larval stage (1074), while the lowest number of proteins were detected in the egg stage (88). The core proteome, i.e., the set of proteins that were consistently quantified across all stages, consisted of 1703 proteins (Fig. 1d). To gain insight into the biological functions of these consistently expressed proteins, we performed gene ontology (GO) enrichment analysis. As expected, the core proteome was enriched for general processes such as protein synthesis and regulation (“cytoplasmic translation”, “translational initiation”, “translation”, “protein folding”), protein degradation and turnover (“proteasome-mediated ubiquitin-dependent protein catabolic process”, “proteasomal protein catabolic process”, “ubiquitin-dependent protein catabolic process”), and intracellular transport (“intracellular protein transport”, “protein import into nucleus”) (Supplementary Data S1).

**Fig. 2:**
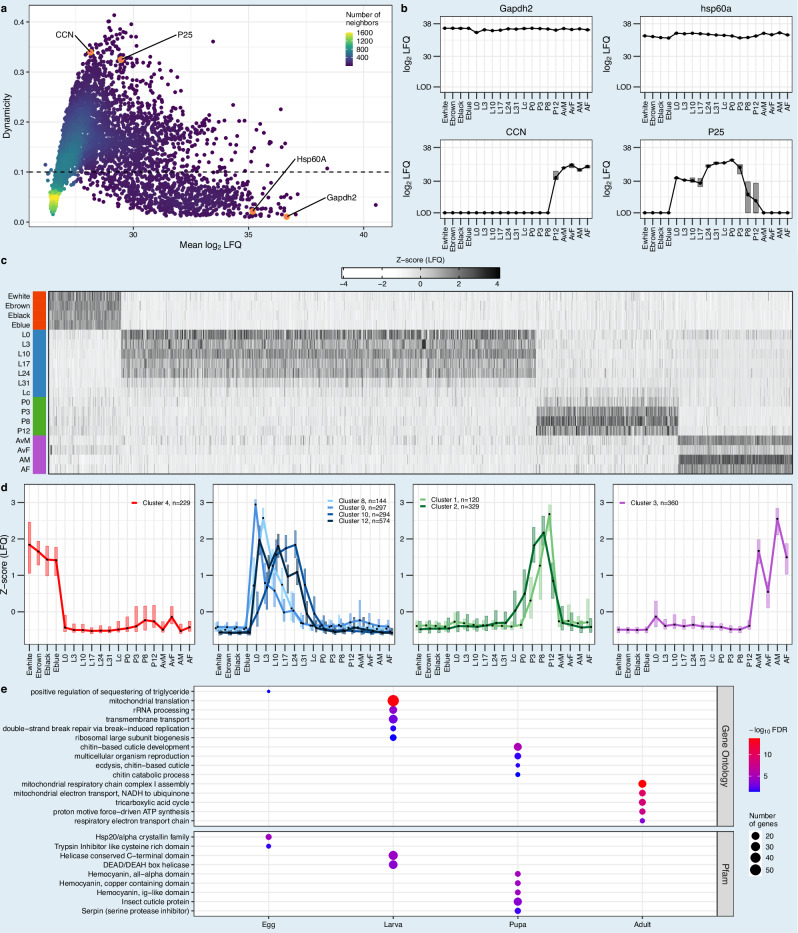
Dynamicity and stage specificity of the developmental proteome of *B. mori.* **a** Scatter plot of the average protein intensities (mean log_2_ LFQ intensity of all timepoints) in relation to their dynamicity across development (Gini ratio). A filter of 0.1 (dashed line) for the dynamicity score divides the proteome into proteins with stable and dynamic expression. In the lower part, proteins with stable expression are shown (*n* = 763), while the upper part contains proteins with dynamic expression (*n* = 1549). **b** Temporal expression profiles (mean log_2_ LFQ intensities, *n* = 5 biological replicates, LOD = limit of detection) of two highly stably (Gapdh2 and Hsp60A) and two highly dynamically expressed proteins (CCN and P25) highlighted in orange in (**a**). Box plots display the expression distribution of the replicates; the horizontal line represents the median and the upper and lower edges of the box depict the interquartile range (IQR). **c** Heatmap of normalized protein expression (z-score of mean LFQ intensity, *n* = 5 biological replicates) of 2347 proteins (proteins within the upper 90th percentile of protein-specific variability, measured by interquartile range (IQR) across timepoints, quantified in >= 3 replicates) corresponding to stage-specific clusters (egg = 229 proteins, larva = 1309 proteins, pupa = 449 proteins and adult = 360 proteins). **d** Protein expression profiles of individual stage-specific clusters generated by unsupervised SOM clustering, plotted as the cluster-wise median z-score normalized mean LFQ intensities (*n* = 5 biological replicates, same data as in (**c**)). Box plots show the expression distribution of proteins cluster-wise; the black dot represents the median and box edges represent the IQR. **e** Gene Ontology (GO) term and Pfam domain enrichment for the proteins associated with the stage-specific clusters (Fisher’s exact test, FDR < 0.05). Each circle represents a GO term or Pfam domain, with the color corresponding to the −log_10_(FDR) and the size representing the number of proteins associated with the respective category. The top five most significantly enriched terms per stage are depicted (all stage-specific terms in Supplementary Data S2 and cluster-specific in Supplementary Data S3).

Next, we assessed the expression dynamics of all proteins across the entire developmental time course using the Gini coefficient (Fig. 2a). Gapdh2 and Hsp60, which perform essential functions and constitute established loading controls^42–45^, show constant expression levels across all developmental stages (Fig. 2b). The protein P25, a major component of silk fibroin^46^, was found in larvae and pupae. This trend can also be seen in our transcriptome data (Supplementary Fig. S4). So far, P25 expression has only been shown in larvae^47^. Cocoonase (CCN), the enzyme necessary to digest the cocoon for hatching^48^, was found only in the late stage of pupae and in adults (Fig. 2b).

To determine more stage-specific proteins, we performed unsupervised clustering using self-organizing maps (SOMs) of the normalized protein intensities of the most dynamically expressed proteins. Overall, 2347 proteins were assigned to stage-specific clusters, representing expression specific to eggs, larvae, pupae and adults (Fig. 2c and Supplementary Fig. S5). While egg- and adult-specific proteins form single clusters, larval- and pupal-specific proteins assemble into multiple clusters (4 and 2 clusters, respectively, Fig. 2d). To understand the functional relevance of the stage-specific expression, we employed Gene Ontology (GO) analysis. To this end, we inferred GO annotations for *B. mori* by utilizing orthologous relationships with *D. melanogaster*. We complemented GO annotations with direct Pfam predictions based on *B. mori* protein sequences to provide species-specific protein family and domain information.

Egg-specific proteins were enriched for the GO term “positive regulation of sequestering of triglyceride”. We also found that the Pfam domain “Hsp20/alpha crystallin family” (Fig. 2e) was enriched in this set of proteins. Proteins of this family mainly act as chaperones, binding to denatured proteins to protect cells from damage^49^. However, they are also involved in other functions, such as protein folding, transport and embryonic development^50^. The enriched GO terms for larvae were mainly related to translation, with “mitochondrial translation” being the most significantly enriched term. These biological processes have previously been demonstrated to be pivotal for feeding and growth in *D. melanogaster* larvae^51^, both of which are characteristic features of holometabolous larvae. In addition, larval-specific proteins are enriched for helicase-related domains. Helicases are closely associated with RNA regulation^52^, which is consistent with the enriched GO terms associated with translation processes. In pupae, the majority of enriched GO terms were related to chitin and cuticle development. One of these terms, “ecdysis”, describes the process of shedding off the old cuticle^53^. The most significantly enriched Pfam domains are hemocyanins, which are associated with oxygen transport^54^ but are also described in storage proteins that are assumed to provide nitrogen and amino acids to pupae and adults during metamorphosis^55^. In the adult stage, the most significantly enriched GO terms predominantly relate to mitochondria and transport or, more specifically, to oxidative phosphorylation (OXPHOS), the central process for energy production. The enriched term “tricarboxylic acid cycle”, essential for OXPHOS, plays a crucial role in generating the reducing equivalents NADH and FADH2^56^. Further enrichment analysis using the Kyoto Encyclopedia of Genes and Genomes (KEGG) database confirmed the significant enrichment of the OXPHOS pathway, with 57 out of 146 proteins showing adult-specific expression (Supplementary Fig. S6).

### Developmental transcriptome of *B. mori* validates the importance of OXPHOS in adults

**Fig. 3:**
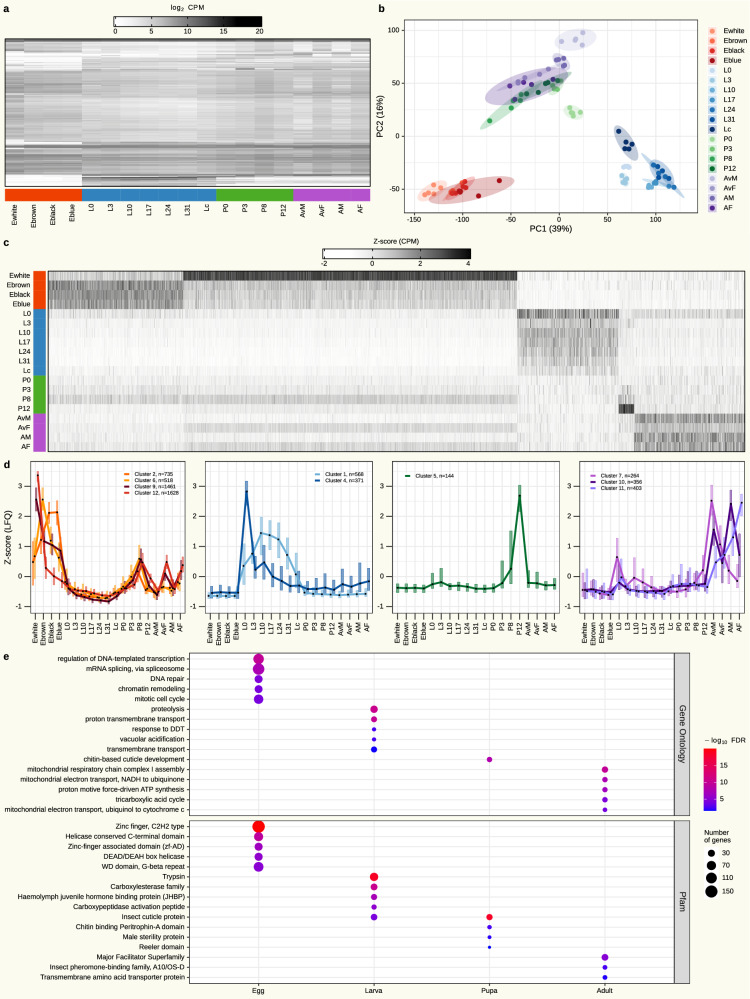
Developmental transcriptome of *Bombyx mori.* **a** Heatmap of average transcript abundance (mean log_2_(CPM + 1) values of the 5064 most variable transcripts (transcripts within the upper 30th percentile of transcript-specific variability, measured by interquartile range (IQR) across timepoints)). **b** Scatter plot of the first two principal components of the dataset shown in (**a**). Biological replicates are depicted in the same color, with elliptical areas representing the standard deviation between replicates. **c** Heatmap of normalized transcript abundance (z-score of mean CPM values) of the 6448 most variable transcripts (transcripts within the upper 90th percentile of transcript-specific variability, measured by IQR across timepoints) corresponding to stage-specific clusters (egg = 4342 transcripts, larva = 939 transcripts, pupa = 144 transcripts and adult = 1023 transcripts). **d** Transcript expression profiles of individual stage-specific clusters generated by unsupervised SOM clustering plotted as the cluster-wise median z-score normalized mean CPM values. Box plots show the expression distribution of transcripts cluster-wise; the black dot represents the median and box edges represent the IQR. **e** Gene Ontology (GO) term and Pfam domain enrichment for the transcripts associated with the stage-specific clusters (Fisher’s exact test, FDR <0.05). Each circle represents a GO term or Pfam domain, with the color corresponding to the −log_10_(FDR) and the size representing the number of transcripts associated with the respective category. The top five most significantly enriched terms per stage are depicted (all terms in Supplementary Data S7). All data is based on *n* = 5 biological replicates, except for L10, L17, L24, and L31, for which *n* = 4.

To obtain further complementary information on the life cycle of *B. mori*, we sequenced poly-A-tailed RNA from identical samples utilized for proteomic measurements. Overall, the raw RNA-seq data included 423 million 3’-end single-end reads, with a mean of 4.6 million reads per library. On average, 57% of the reads mapped to *B. mori* open reading frames, resulting in 14,750 transcripts with assigned reads in any sample (Supplementary Fig. S7). The expression levels of the most variable transcripts differed between stages (Fig. 3a). To assess the quality of the data, we determined Pearson correlations and obtained a high reproducibility between replicates (Pearson’s *R* = 0.8–1) of the same timepoints (Supplementary Fig. S8). For the first two PCA components, the replicates clustered closely together. While egg and larval stages clustered separately, pupa and adult stages were intermingled (Fig. 3b). Interestingly, white eggs (Ewhite), the earliest timepoint, clustered separately from the other egg timepoints. This is also evident in the correlation heatmaps of both transcriptome and proteome samples (Supplementary Figs. S8 and S3, respectively). As the fusion of sperm and egg pronuclei occurs within a few hours after oviposition^57^, the transcripts and proteins detected in Ewhite are most likely maternally deposited. The observed shift in expression between Ewhite and brown eggs (Ebrown) may reflect the onset of zygotic transcription following fertilization. To further investigate potential maternal genes, we cross-referenced our dataset with 1534 previously established maternal genes in *Drosophila melanogaster*^58^. Notably, among the 2101 transcripts significantly more abundant in Ewhite compared to Ebrown (FDR <0.05, fold change >2; Supplementary Data S4), we identified a significant enrichment of *D. melanogaster* maternal genes (*P* = 0.0009, one-tailed Fisher’s exact test). However, at the proteome level, no such enrichment was observed (*P* = 0.15, one-tailed Fisher’s exact test; Supplementary Data S5), which is expected taking into consideration that maternal genes are predominantly deposited as mRNAs rather than proteins^59^. A functional enrichment analysis of transcripts significantly more abundant in Ewhite than in Ebrown, representing potential *B. mori* maternal transcripts, revealed an overrepresentation of DNA metabolism-related functions (Supplementary Data S6). This finding is consistent with previously reported functions of maternal genes^58^. Collectively, these results suggest that the earliest two timepoints, Ewhite and Ebrown, may capture the transition from maternal to zygotic gene expression.

As for the proteome analysis, we were interested in stage-specific transcript expression. For this purpose, the most variably expressed transcripts were clustered by an artificial neural network algorithm and filtered for stage-specific expression profiles (Fig. 3c and Supplementary Fig. S9). While pupal-specific transcripts formed a single cluster, egg-, larva-, and adult-specific transcripts assembled into multiple clusters (4, 2 and 3 clusters, respectively) (Fig. 3d). We performed functional enrichment of these stage-specific transcript groups to identify relevant biological processes overrepresented in these developmental stages (Fig. 3e). Egg-specific expression was significantly associated with GO terms related to genome maintenance and regulation. The most significantly enriched biological process term “regulation of DNA-templated transcription” should be important in embryonic development, as transcriptional regulation governs the precise timing and coordination of gene expression^60^. Consistent with this, we also found highly significant enrichment of zinc finger- and helicase-associated Pfam domains. Zinc fingers are diverse proteins involved in various critical functions, including DNA recognition and transcriptional activation, which are vital for early embryonic development^54^. Moreover, helicases, particularly those in the DEAD-box helicase family, play crucial roles in post-transcriptional RNA regulation, ensuring precise gene expression during early embryogenesis^61^. Interestingly, egg-specific clusters could be further divided into two main profiles (Fig. 3d). Clusters showing high initial levels with a steady decrease in transcript abundance (dark red: clusters 9 and 12) were enriched for cell division-related functions such as “DNA replication”, “centrosome cycle”, and “histone acetylation”, consistent with the intense cell proliferation that occurs during early embryonic development. Clusters with later transient expression peaks (light red: clusters 2 and 6) are associated with terms related to transposable element (TE) regulation. Such transient TE expression has previously been described in *D. melanogaster* and is believed to be important for regulating zygotic gene activation^62^ (all terms in Supplementary Data S8).

In *B. mori*, larvae are the primary feeding stage, with the midgut serving as the central site for digestion and nutrient absorption. In this context, the enrichment of “proteolysis” could be related to digestive enzymes responsible for protein digestion. In addition, the terms “vacuolar acidification” and “proton transmembrane transport” might be involved in regulating the pH environment within the midgut for optimal digestive enzyme activity and nutrient absorption^63^. Consistent with the above, we observed enrichment of the Pfam domain “Trypsin”, which is found in trypsin family members consisting of protease enzymes that play a key role in proteolysis. In addition, the Pfam “Carboxylesterase family” is important for the breakdown of food sources and detoxification^64^. Interestingly, the two larval clusters also showed significant differences. Cluster 1 expression exhibited bell-shaped dynamics, with an initial increase, a peak in the middle and a subsequent decrease. In contrast, cluster 4 peaks in the first larval stage with considerably lower expression levels at later timepoints. Both clusters were enriched for digestion-related functions.

For the pupal stage, expression levels of stage-specific genes peak shortly before hatching at P12. The only enriched GO term was “chitin-based cuticle development”, which aligns with the most enriched Pfam domain, “Insect cuticle protein". This Pfam domain is enriched in both larvae and pupae, stages in which molting occurs and a new cuticle is formed. Another enriched Pfam domain “Chitin binding Peritrophin-A domain” is predominantly associated with peritrophic membrane proteins and has been shown to be involved in chitin binding. Experimental evidence indicates that proteins containing this domain are crucial for the formation of a new cuticle during molting and metamorphosis^65^.

In the adult stage, we obtained three different expression clusters, with transcripts in cluster 7 peaking in adult virgin males, cluster 10 showing generally higher expression in males and cluster 11 gradually increasing, with the maximum levels in adult females (5 days). The predominantly enriched GO terms for adult-specific transcript expression were related to OXPHOS. This mirrors the significantly enriched OXPHOS-related GO terms previously identified in the adult-specific proteome (Fig. 2e), demonstrating coherence of transcript and protein expression of this pathway in adults. The most significantly enriched Pfam “Major facilitator superfamily” is one of the largest membrane transporter families. Proteins within this family play a central role in the cellular transport of nutrients, sugars and amino acids^66^. The “Insect pheromone-binding family, A10/OS-D” domain is associated with insect pheromone-binding proteins, which are essential for pheromone transport and recognition, enabling the localization of mates and facilitating mating^67^.

### The transcript and protein levels of most *B. mori* genes do not correlate throughout development

We identified OXPHOS as an important process in the adult stage of *B. mori* supported by transcriptome and proteome analysis. To further explore the resemblance between the two expression levels, we systematically compared the genes included in the transcriptome and proteome clusters. Generally, there was stage-wise coherence between these clusters, exhibiting more significant overlap between clusters of the same stages (Supplementary Fig. S10). For instance, the same expression profiles with very significant overlap among their genes were apparent for the adult-specific clusters – transcriptome cluster 10 and proteome cluster 3 (FDR < 10^−25^, Fisher’s exact test) – and the larval-specific clusters – transcriptome cluster 1 and proteome cluster 10 (FDR < 10^−72^, Fisher’s exact test). However, there are also differences in the stage-specific expression of the transcriptome and proteome. The number of stage-specific genes varied between these two gene regulatory layers: 56% (1309 proteins) of the stage-specific proteins were found in larvae, while 67% (4342 transcripts) of stage-specific transcripts were expressed in the egg. Surprisingly, there was a significant overlap (FDR <10^−15^, Fisher’s exact test) in the genes in egg-specific transcriptome cluster 9 and larval-specific proteome cluster 12, which might indicate that the mRNAs of some genes are synthesized in the egg stage for later translation in the larvae. This set of genes was enriched for GO terms associated with RNA regulation and processing.

**Fig. 4:**
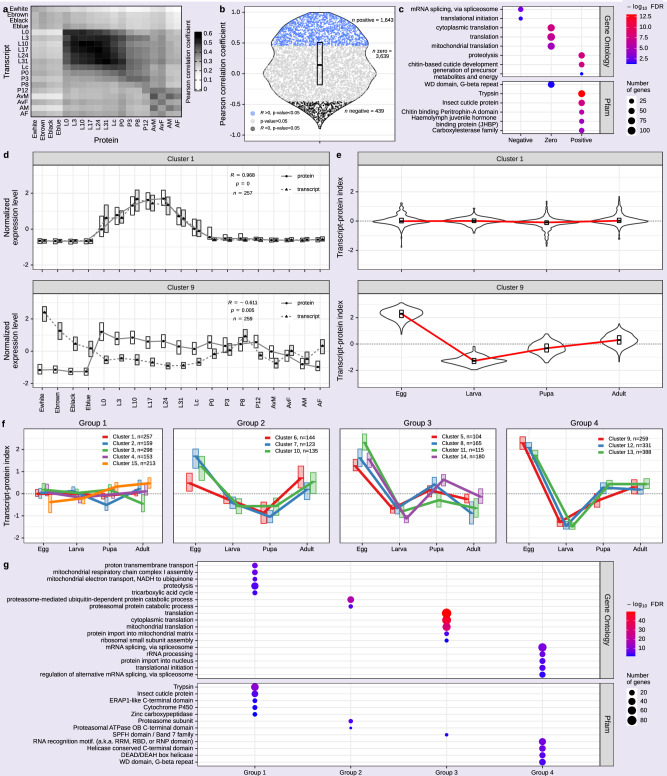
Global and stage-specific patterns of transcript–protein correlations across *Bombyx mori* development. **a** Heatmap displaying the Pearson correlation coefficient between transcript and protein expression (*n* = 6032 genes, quantified in transcriptome and proteome). **b** Violin plot showing the distribution of Pearson correlation coefficients between transcript and protein abundance per gene (*n* = 5271 genes with dynamic expression at either the transcript and/or protein level). Black, blue and gray dots represent negative (Pearson’s *R* < 0, *P* value < 0.05), positive (Pearson’s *R* > 0, *P* value < 0.05) and no transcript–protein correlation (*P* value ≥ 0.05), respectively. The box plot displays the distribution of Pearson’s *R*; the horizontal line represents the median and the upper and lower edges of the box depict the interquartile range (IQR). **c** Significantly enriched Gene Ontology (GO) terms and Pfam domain results of genes with negative, no (zero) and positive transcript–protein correlation (Fisher’s exact test, FDR < 0.05). Color corresponds to enrichment −log_10_(FDR), and the circle size represents the number of genes per GO term or Pfam domain. The top 5 most significantly enriched terms with negative, no and positive transcript–protein correlations are depicted (all terms in Supplementary Data S9). **d** Protein (dashed line) and transcript (solid line) expression levels of genes assigned to the respective clusters (cluster-wise median z-score of mean CPM values and mean LFQ values) with highly positive (cluster 1) or negative (cluster 9) transcript–protein correlations generated by unsupervised SOM clustering are shown. The box plots show the distribution of normalized expression levels; black dot or triangle represent the median and box edges indicate the IQR. In addition, for each cluster, the Pearson’s *R* between protein and transcript expression levels and the corresponding *P* value are depicted. **e** Violin plots illustrating the stage-specific transcript–protein index (representing the difference in means between transcript and protein expression). Box plots display the transcript–protein index distribution; the horizontal line represents the median and the upper and lower edges of the box depict the IQR. The red line connects stage-specific median transcript–protein indexes. **f** Line plots displaying the median stage-specific transcript–protein indexes of the 15 clusters assigned to 4 groups using k-means clustering. Box plots show the distribution of transcript–protein indexes, the horizontal lines represent the median, and the box edges indicate the IQR. **g** Significantly enriched GO term and Pfam domain results (Fisher’s exact test, FDR < 0.05) for genes within each of the four groups depicted in (**f**). Color corresponds to enrichment −log_10_(FDR), and the circle size represents the number of genes per GO term or Pfam domain. The top five most significantly enriched terms per group are depicted (all terms in Supplementary Data S10).

To further exploit our paired transcriptome–proteome data, we explored the temporal correlation of transcriptome and proteome across development in more depth. Assessing timepoint-wise correlations between both levels revealed better coherence within the stages (Fig. 4a), apparent as a diagonal correlation pattern with the larval stage showing the highest correlations (mean Pearson’s *R* = 0.5), while eggs exhibited overall lower correlations (mean Pearson’s *R* = 0.29). To trace transcript–protein correlations in a gene-wise manner, we selected all genes with dynamic expression patterns in at least one of the sets (5721 genes). The transcriptomic and proteomic data of these genes were used to determine correlations between the two levels of expression. Of these, 1643 proteins (29%) showed positive correlation (Pearson’s *R* > 0, *P* value < 0.05) with their transcript expression (Fig. 4b). However, most proteins (3639 or 64%) showed no correlation with their transcript expression levels (*P* value ≥0.05). A total of 439 (8%) proteins exhibited negative transcript–protein correlations (Pearson’s *R* < 0, *P* value < 0.05). To explore potential functional characteristics of genes with positive, no or negative transcript–protein expression correlations, we performed functional enrichment analysis (Fig. 4c). Positively correlated genes were enriched for the GO term “proteolysis”. Proteolysis is involved in essential cellular functions, such as enzyme activation and deactivation, protein quality control and regulation of various physiological processes^68^. Similarly, the most enriched Pfam domain was “Trypsin”, a protease family important in proteolysis. In addition, genes with such positive transcript–protein correlations were enriched for the GO term “chitin-based cuticle development” and Pfam domains “Insect cuticle protein”, and “Chitin binding Peritrophin-A domain”, all of which are related to cuticle formation. Notably, similar terms were enriched in our stage-specific transcript (Fig. 3e) and protein clusters (Fig. 2e). Another enriched Pfam, “Haemolymph juvenile hormone binding protein (JHBP)” refers to proteins responsible for transporting and protecting juvenile hormones (JH) in the haemolymph. Juvenile hormones (JH) regulate many processes in insects, including development, metamorphosis, and reproduction^69^. Interestingly, high transcript–protein correlations are mostly found in processes linked to physiological changes and stage-specific regulated gene expression. Genes with no significant transcript–protein expression correlation were associated with “mitochondrial translation”, “cytoplasmic translation”, and translation, while genes with negative transcript–protein correlation were enriched in GO terms such as “mRNA splicing” and “translational initiation”. This may illustrate more post-transcriptional gene regulatory control of these functions during development.

To explore underlying developmental dynamics, we applied unsupervised SOM clustering using a neural network method on both the transcriptome and proteome data in parallel, yielding 15 clusters (Supplementary Fig. S11). Transcript and protein expression levels were closely correlated for cluster 1 (Pearson’s *R* = 0.973, Fig. 4d), while there was an overall negative correlation for genes in cluster 9 (Pearson’s *R* = −0.585, Fig. 4d). Examining the transcript–protein expression differences for all 15 clusters (Supplementary Fig. S11), we observed stage-specific trends. In most low-correlation clusters, the differences in protein and transcript expression seem to be rather stage-specific and are most pronounced in the egg and larval stages. Hence, correlations across all timepoints were only partially informative. We thus compared transcript and protein expression stage-wise for each cluster (Fig. 4e and Supplementary Fig. S12). As anticipated, clusters with high overall correlation between transcript and protein expression exhibited minimal stage-specific differences (Fig. 4e, cluster 1), as indicated by a transcript–protein index (a measure of the difference in means, i.e., mean normalized transcript expression minus mean normalized protein expression) of approximately zero. Conversely, clusters with low correlation showed diverse patterns of stage-specific transcript–protein differences. For example, in cluster 9, high transcript and low protein expression in the egg stage corresponded to a high transcript–protein index. However, during the larval stage, this trend reversed to higher protein and lower transcript expression, resulting in a negative transcript–protein index (Fig. 4e, cluster 9). Determination of such stage-specific transcript‒protein indexes is necessary for better resolved temporal patterns of transcript–protein dynamics. To this end, we grouped clusters based on their stage-specific transcript–protein index dynamics and obtained four groups with similar transcript–protein homeostasis across stages (Fig. 4f). Group 1 comprised five clusters (4320 genes) with similar transcript–protein dynamics (transcript–protein indexes of approximately zero) across all stages. Proteins within this group are linked to GO terms associated with oxidative phosphorylation (Fig. 4g), such as “mitochondrial respiratory chain complex I assembly”, “mitochondrial electron transport, NADH to ubiquinone”, and “proton transmembrane transport”. Consistent with the functional characterization of the genes with positive gene-wise transcript–protein correlations (Fig. 4b, c), group 1 also exhibited enrichment of the GO term “proteolysis” and the corresponding Pfams “Trypsin” and “Insect cuticle protein”. Group 2 (3 clusters, 1608 genes) was characterized by positive transcript–protein indexes in the egg and adult stages, i.e., high transcript levels but relatively low protein expression in these stages, while we observed higher relative protein levels in the pupal stages. Both enriched GO terms and Pfam domains are associated with proteasomal protein catabolism.

Groups 3 and 4 showed similar characteristics, with relatively high transcript levels in the egg stage (high transcript–protein index) and relatively high protein levels in the larval stage (negative transcript–protein index). This effect is more pronounced for the clusters of group 4 (3 clusters, 3912 genes). While protein and transcript changes were similar during the adult stage in group 4, group 3 (4 clusters, 2256 genes) showed slightly negative transcript–protein indexes in adults. Highly enriched GO terms for group 3 were “cytoplasmic translation” and “mitochondrial translation”, and those for group 4 were “mRNA splicing via spliceosome” and “rRNA processing”. Hence, these two groups are functionally associated with RNA and post-transcriptional regulation. Consistently, the enriched Pfam domains in group 4, “RNA recognition motif (a.k.a. RRM, RBD, or RNP domain)” and two domains linked to helicases, are associated with proteins that regulate various processes of post-transcriptional gene expression^52,61,70^.

In summary, our integrative analysis detected two sets of genes, such that have coherent transcript and protein dynamics throughout development (group 1) and those that show distinct expression changes at the two layers (groups 2, 3, and 4). The majority of the differentially regulated genes are associated with RNA regulation and translation, suggesting that these cellular functions may be more post-transcriptionally controlled.

### Comparative developmental systems biology in *B. mori* and *D. melanogaster*

*Drosophila melanogaster* (fruit fly), one of the most extensively studied model organisms, is like *B. mori* a holometabolous insect which undergoes complete metamorphosis throughout its life cycle, exhibiting the same developmental stages (i.e., egg, larva, pupa, and adult). A comparison of *B. mori* and *D. melanogaster* therefore enables the identification of common gene regulatory features but also reveals species-specific differences among holometabolous insects. For this, we relied on orthologous gene relationships between both species established by SonicParanoid^71^. We used the developmental proteome of *D. melanogaster*^12^ to compare both species. To focus on dynamic processes across the life cycle, only the most variable proteins in any of the species were analyzed. For a general overview, we correlated the proteome data of the two species, taking all possible pairs of timepoints into account (Supplementary Fig. S13a). Overall, the two datasets exhibit greater coherence in most corresponding stages, with a mean correlation coefficient of 0.56 across stage comparisons. Interestingly, adult timepoints showed the greatest similarity (mean Pearson’s *R* = 0.63), while eggs were more distinct overall (mean Pearson’s *R* = 0.44). Notably, *B. mori* larval timepoints were also highly similar to *D. melanogaster* adult timepoints (mean Pearson’s *R* = 0.61).

**Fig. 5:**
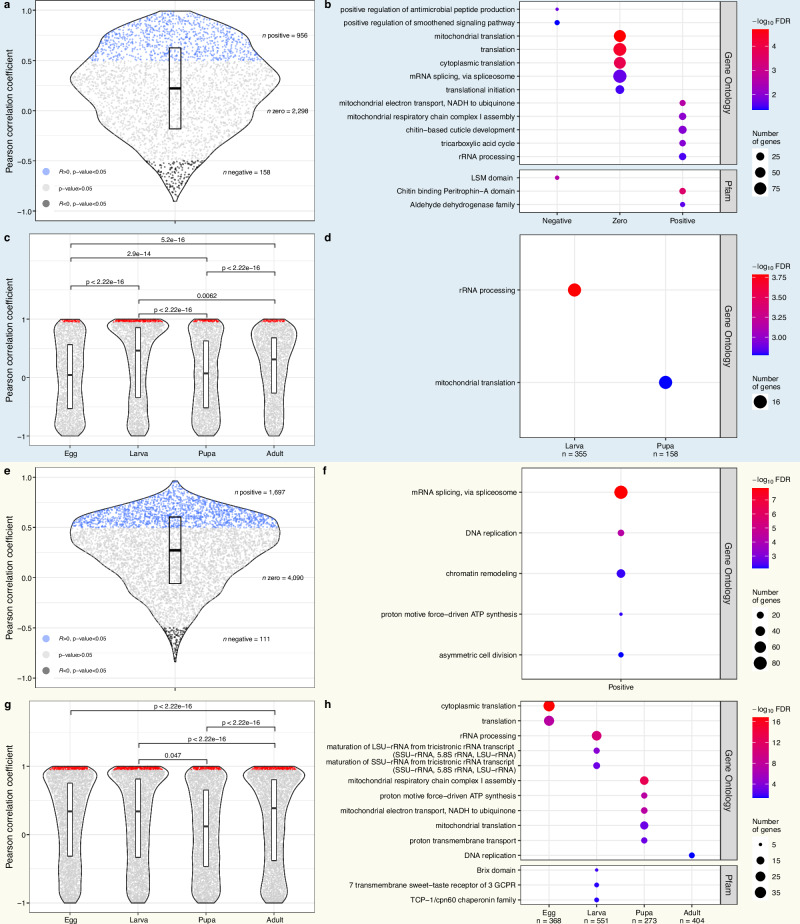
Comparative analysis of protein and transcript dynamics between *Bombyx mori* and *Drosophila melanogaster.* **a** Violin plot showing the distribution of Pearson correlation coefficients between the protein abundance of *B. mori* and *D. melanogaster* orthologs (proteins within the upper 70th percentile of protein-specific variability, measured by interquartile range (IQR)) across timepoints (*n* = 3412 orthologs with dynamic expression in at least one species). Black, blue and gray dots represent negative (Pearson’s *R* < 0, *P* value < 0.05), positive (Pearson’s *R* > 0, *P* value < 0.05) and no protein correlation (*P* value ≥ 0.05) between both species, respectively. The box plot displays the Pearson’s *R* distribution; the horizontal line represents the median, and the upper and lower edges of the box depict the IQR. **b** Significantly enriched gene ontology (GO) terms and Pfam domain results (Fisher’s exact test, FDR < 0.05) for orthologs with negative, no (zero) and positive protein correlations between both insects. Color corresponds to enrichment −log_10_(FDR), and the circle size represents the number of genes per GO term or Pfam domain. The top five most significantly enriched terms per negative, no (zero) and positive protein correlation are depicted (all terms in Supplementary Data S12). **c** Violin plots showing distributions of stage-specific Pearson correlation coefficients between the protein levels of *B. mori* and *D. melanogaster* orthologs across all timepoints (egg *n* = 3142, larva *n* = 3275, pupa *n* = 3246, adult *n* = 3283). The box plot displays the Pearson’s *R* distribution; the horizontal line represents the median, and the upper and lower edges of the box depict the IQR. **d** Significantly enriched GO term and Pfam domain results of highly correlated orthologs per stage (highlighted red in c). Plot design parameters are the same as in (**b**) (all terms in Supplementary Data S13). **e** Violin plot showing the distribution of Pearson correlation coefficients between the transcript abundance of *B. mori* and *D. melanogaster* orthologs (transcripts within the upper 70th percentile of transcript-specific variability, measured by IQR) across all timepoints (*n* = 5898 orthologs with dynamic expression in at least one species). Black, blue and gray dots represent negative (Pearson’s *R* < 0, *P* value < 0.05), positive (Pearson’s *R* > 0, *P* value < 0.05) and no transcript correlation (*P* value ≥ 0.05) between both species, respectively. The box plot displays the Pearson’s *R* distribution; the horizontal line represents the median, and the upper and lower edges of the box depict the IQR. **f** Significantly enriched GO terms and Pfam domain results (Fisher’s exact test, FDR < 0.05) for orthologs with negative, no (zero) and positive transcript correlations between both insects. Color corresponds to enrichment −log_10_(FDR), and the circle size represents the number of genes per GO term or Pfam domain. The top five most significantly enriched terms per negative, no (zero) and positive transcript correlation are depicted (all terms in Supplementary Data S14). **g** Violin plots showing distributions of stage-specific Pearson correlation coefficients between the transcript levels of *B. mori* and *D. melanogaster* orthologs across all timepoints (egg *n* = 5671, larva *n* = 5812, pupa *n* = 5772, adult *n* = 5730). The box plot displays the Pearson’s *R* distribution; the horizontal line represents the median, and the upper and lower edges of the box depict the IQR. **h** Significantly enriched GO term and Pfam domain results of highly correlated orthologs per stage (highlighted red in (**g**)). Plot design parameters are the same as in (**f**) (all terms in Supplementary Data S15).

Investigating the developmental expression patterns of 3412 orthologs individually, 956 proteins (28%) exhibited significant positive cross-species protein expression correlations (*P* value < 0.05, Pearson’s *R* > 0, Fig. 5a). The vast majority of orthologs (2298 or 67%) showed no correlation (*P* values ≥ 0.05), while a small fraction of 158 orthologs (5%) showed significantly negative correlations (*P* value < 0.05, Pearson’s *R* < 0).

To characterize common and distinct features between positively, non-correlated and negatively correlated orthologs, we performed functional enrichment analysis (Fig. 5b). The two most significantly enriched GO terms associated with orthologs that exhibit similar expression dynamics are related to mitochondrial processes. Specifically, they are both associated with complex I in the oxidative phosphorylation pathway. Notably, the processes and components of the OXPHOS pathway have been shown to be highly conserved across even more distant species^72^. Expression dynamics of the OXPHOS complex I-associated orthologs were similar, with the highest expression levels occurring in adults of both species (Supplementary Fig. S14). The enriched Pfam domain “Chitin binding Peritrophin-A domain” is mainly linked to peritrophic membrane proteins and is involved in chitin binding. Experimental evidence suggests the domain’s involvement in the formation of a new cuticle during molting and metamorphosis in insect development^65^. The positive correlation could be attributed to both insects undergoing full metamorphosis with shared stages, suggesting a common need for these proteins during developmental transitions. Another enriched Pfam was “aldehyde dehydrogenase family” encompassing proteins involved in detoxification processes in *D. melanogaster*^73^.

The three most significantly enriched GO terms associated with orthologs that have distinct protein expression dynamics in the two insects are all related to the regulation of translation. Interestingly, GO terms related to “translation” were also enriched in genes with distinct transcript–protein dynamics in *B. mori* (Fig. 4c). The GO term “positive regulation of antimicrobial peptide production” was enriched among proteins with distinct protein dynamics in the two species. Antimicrobial peptides are pivotal elements of the innate and adaptive immune system and are critical in the defense against microbial pathogens^74^. Analysis across the entire life cycle could mask proteome similarities that are restricted to certain stages, and we thus examined protein expression between the two species for each main developmental stage (egg, larva, pupa, and adult) individually.

Upon examining the distribution of the correlation coefficients between the protein expression levels of the two insects stage-specifically, we observed that adult and especially larval correlations were generally higher (Fig. 5c). We performed functional enrichment analysis for the orthologs that showed a significantly high correlation in the individual stages (Pearson’s *R* > 0, *P* value < 0.05) (egg = 95, larva = 355, pupa = 158, and adult = 118). Among these, only two GO terms were enriched, “rRNA processing” for larvae and “mitochondrial translation” for pupae, both constituting basic cellular processes (Fig. 5d).

In addition to the comparative proteome analysis, we also compared the developmental transcriptome dynamics of the most variable transcripts between both species. For this, we used previously published transcriptome data covering the entire life cycle of *D. melanogaster*^4^. For a general overview, we correlated the transcriptome data of the two species, considering all possible pairs of timepoints (Supplementary Fig. S13b). The developmental transcriptomes of the two species displayed high coherence in egg (mean Pearson’s *R* = 0.49), larval (mean Pearson’s *R* = 0.54) and adult stages (mean Pearson’s *R* = 0.47), whereas pupae exhibited overall lower correspondence (mean Pearson’s *R* = 0.41), with a mean correlation coefficient of 0.48 across all stage comparisons. Interestingly, the two species display less similarity at the transcript level than at the proteome level, except for the egg stage, which exhibits higher transcript correlations.

We determined individual transcript expression correlations between the two insects across the life cycle. Among the 5898 orthologs, 1697 (29%) showed positive transcript expression correlations, around 70% of the orthologs (4090) exhibited no correlation, and a small number of 111 orthologs (2%) showed negatively correlated transcript expression during development (Fig. 5e). The most significantly enriched GO terms for orthologs with positive transcript correlations were “mRNA splicing, via spliceosome” and “DNA replication” (Fig. 5f), representing essential cellular functions^75,76^.

Transcript expression correlations between the two insects were also calculated for the four developmental stages individually. Overall, egg, larval and adult stages showed significantly higher stage-specific correlations than pupal stage (Fig. 5g). Functional enrichment analysis of the most highly correlated orthologs in the individual stages (R > 0, *P* value < 0.05) (egg = 368, larva = 551, pupa = 273, and adult = 404) revealed that the GO terms “cytoplasmic translation” and “translation” were highly enriched in the egg stages, indicating their importance during early development in both insects (Fig. 5h). In the larval stage, enrichment was observed for terms related to rRNA processing and maturation. In accordance with this result, the most enriched Pfam domain “Brix domain”, is associated with proteins involved in rRNA processing and ribosome biogenesis^77^. The larval stage is characterized by an immense increase in body size, and ribosomal biogenesis seems to be particularly important for the regulation of larval growth^78^. Interestingly, rRNA processing was associated with both highly correlated transcripts and proteins in the larval stage (Fig. 5h, d). The majority of highly enriched GO terms in pupae were related to oxidative phosphorylation. Examining expression profiles of the genes associated with the three most significantly enriched GO terms, a clear trend of increasing expression toward later pupal stages was observed (Supplementary Fig. S15). In addition, similar to protein correlation, “mitochondrial translation” is an enriched GO term for highly correlated transcript patterns in the pupal stages. The only functionally enriched GO term for the adult stage was “DNA replication”.

We compared the proteome and transcriptome data of *B. mori* and *D. melanogaster* in a gene-wise manner for the whole life cycle and for individual stages. In general, proteome and transcriptome comparisons across the two insects revealed similar correlations and percentages of positively correlated genes/proteins. However, overall, there seem to be greater similarities in the proteome comparisons (Fig. 5a, e). The stage-specific analysis revealed that the larva exhibited the greatest coherence between orthologs in both the transcriptome and proteome datasets (Fig. 5c, g).

## Discussion

Our data represents the first developmental proteome including all major stages throughout the entire life cycle of the silkworm *B. mori*. In addition, we sequenced the transcriptomes of the same samples to enable direct comparison of these two levels of regulation. By exploring and comparing these datasets, we were able to uncover interesting underlying mechanisms that might be crucial for developmental processes in *B. mori*. The comprehensive datasets serve as a resource for future research in silkworms and for comparisons with other insects. Similar to *B. mori*, the fruit fly is a holometabolous insect that undergoes complete metamorphosis throughout its life, with the same stages (egg, larva, pupa, and adult). Adding an evolutionary angle, we also compared our *B. mori* data with the previously published developmental proteome and transcriptome of *D. melanogaster*^4,12^, revealing both common gene regulatory features and species-specific differences.

Our developmental proteome of *B. mori* encompasses expression levels of 6058 proteins, representing approximately one-third of the 16,880 genes annotated in SilkBase^79^. The highest number of measured proteins was detected for the larval stage (4893 proteins, Fig. 1d). The number of proteins quantified in eggs was lower than in other stages (1979 proteins, Fig. 1d), but is in a similar range as previously reported in eggs^80,81^. The low number may be partially because large portions of the egg consist of nutrient proteins such as yolk proteins that are important for egg development^82,83^. Indeed, the three major yolk proteins, vitellogenin, egg-specific protein and 30 kDa proteins^84^, showed high expression levels, exceeding the upper 99th percentile across all replicates of the different egg timepoints. Overall, protein level distributions slightly shifted to more abundant proteins in eggs (Supplementary Fig. S16), suggesting that the detection of very lowly abundant proteins could have been hindered by the limited dynamic range. We nevertheless identified 229 proteins with egg-specific expression together with ~1700 proteins that could be identified in all stages, including eggs. In addition to these egg-specific biological factors, there are also detection limitations of mass spectrometry measurements that generally apply to all samples. Combined with our approach of extracting material from whole specimens, proteins with low abundance or those expressed in only small subpopulations of cells are likely to be missed by this method. Consequently, non-identified proteins may either not be expressed or may be expressed below our limit of detection (LOD) enforced by mass spectrometry measurements. In addition, our study focused on silkworms reared under standard conditions, potentially excluding stress-related or condition-specific proteins from our analysis.

Using the same samples, we also generated an extensive transcriptome dataset of 17 timepoints during *B. mori* development and detected 14,750 transcripts covering almost 90% of SilkBase annotated genes. The identified numbers of transcripts were evenly distributed across all timepoints, with a mean of 7413 detected transcripts (Supplementary Fig. S7). Similar expression patterns as those observed in the stage-specific transcriptome clusters (Fig. 3d) were also previously observed in the holometabolous insect *Tribolium castaneum*^85^. Remarkably, our egg transcriptomes included 4342 stage-specifically expressed genes, which represented 67% of all stage-specific genes (Fig. 3d), a characteristic that is not reflected in the proteome data for which most stage-specifically expressed proteins correspond to larvae (1309 proteins, 56%, Fig. 2d). Focusing on stage specificity for both transcriptome and proteome, we detected functional enrichments that were highly consistent with developmental traits of the respective stages. We observed most striking similarities in adult-specific expression between the transcriptome and the proteome, with both being enriched with GO terms associated with the oxidative phosphorylation (OXPHOS) pathway. These include subunits of all major OXPHOS complexes, suggesting major importance of this pathway in the adult stage (Supplementary Fig. S6). There could be several potential reasons, such as the increased energy requirements associated with the use of flight muscles during mating. Flight muscles in *D. melanogaster* are highly enriched in mitochondria and have been used for studying oxidative phosphorylation complex assembly^86,87^. Consistent with this, OXPHOS gene expression is significantly elevated in adult thorax samples according to previously published tissue-specific transcriptome data^88^ (Supplementary Fig. S17). The thorax is predominantly composed of flight muscles, further strengthening the connection between increased OXPHOS expression in adults and enhanced wing movement. Interestingly, normalized transcript and protein expression levels of the adult-specific clusters were much higher in males than in females (Figs. 2d and 3d). Although *B. mori* is flightless, male silk moths perform a mating dance involving vigorous wing vibration^89^. This is consistent with previous observations that *B. mori* males have greater flight muscle mass than females^90^. In addition, higher mitochondrial efficiency in females, observed across a range of phyla^91,92^, might need to be compensated in males by further increasing the number of OXPHOS complexes. Notably, *D. melanogaster* mutants with mitochondrial deficiency show male courtship deficits^93^. An additional reason for the highest levels of mitochondrial OXPHOS-associated genes in the last stages (AM, AF, 5 days old) could be aging. Studies in insects, particularly *D. melanogaster*, have shown age-related changes in mitochondrial structure and function. Reduced activity of the respiratory enzyme cytochrome c oxidase and apoptotic cell death have been observed in the flight muscles of aged *D. melanogaster*^94,95^. A previous study reported a reduction in mitochondrial respiration and electron transport in aged *D. melanogaster*^96^. Similar observations were made in vertebrates, where the capacity for oxidative phosphorylation was reduced in older rats, while the mitochondrial membrane potential remained stable^97^. This could be compensated by the increased expression of OXPHOS-related genes in adults.

We used our paired proteome and transcriptome data to determine the overall correlation between both datasets across all timepoints. Protein expression has been shown to lag behind transcript expression in highly time-resolved studies^12,23^, but as our time course spans approximately 45 days, with days between timepoints, this delay might not be reflected in our data. Indeed, the clustering revealed a pronounced diagonal pattern, reflecting greater similarity between transcriptome and proteome at the same timepoints, with a mean Pearson correlation coefficient of 0.42 (Fig. 4a). This moderate correlation is reminiscent of transcript–protein comparisons in previous studies across different species^11–14,98^. This could be influenced by various biological parameters, such as post-transcriptional and post-translational modifications, translation efficiency, different half-lives and degradation rates, which introduce complex dynamics between mRNA and protein levels^10,13,99^. Although technical differences between mRNA and protein measurement techniques may influence mRNA–protein abundance analysis, almost 30% of the genes exhibited highly significant correlations between mRNA and protein levels across the full time course (Fig. 4b). Investigating the mRNA–protein dynamics per developmental stage, we found that protein abundance of the OXPHOS pathway, in contrast to proteins encoding RNA-binding and post-transcriptional gene regulatory proteins, can be mostly explained by mRNA levels (Fig. 4f, g).

We further compared our *B. mori* data with the previously measured developmental transcriptome and proteome of *D. melanogaster*^4,12^. Direct temporal comparison between the two insects is challenging because the generation time of *B. mori* is four times longer than that of *D. melanogaster*^30,100^. *B. mori* has five larval instars, while *D. melanogaster* has three instars resulting in different developmental durations and endocrinological regulation^101^. For better comparison, the collection of silkworm larvae was not synchronized according to the molting cycle, hence gene expression changes associated with molting cannot be compared directly. Despite this challenge, the timepoint-wise expression comparison revealed a general congruency for corresponding stages, with a modest overall correlation of 0.56 for the proteome and a lower correlation of 0.48 for the transcriptome (Supplementary Fig. S13). Interestingly, the adult transcriptome comparison revealed a sex-specific effect, with greater transcriptome similarity between females than between males across both species. This effect could not be detected in the proteome.

In the protein-wise expression comparison, proteins with distinct protein dynamics in the two species were associated with antimicrobial peptides, which are key components of the innate and adaptive immune system and play crucial roles in defense against microbial pathogens^74^ (Fig. 5b). The negative correlation may reflect adaptations to distinct biotic environments occupied by the two insects. During the immobile pupal stage, defense mechanisms are particularly important. Therefore, an additional contributing factor could be the contrasting protective strategies employed. Silkworms are enclosed and protected by a cocoon, while fruit fly pupae are protected by the puparium, which is a hardened larval cuticle. Although both provide protection, their distinct nature may account for variations in antimicrobial peptide expression. Orthologs with similar developmental protein dynamics in both insects are associated with the oxidative phosphorylation pathway (Fig. 5b). The expression of these orthologs peaked in adults in both species (Supplementary Fig. S14). Notably, this is consistent with the observed adult-specific protein expression in *B. mori* and suggests a potential association with increased energy demands, possibly due to flight muscle utilization and engagement in mating behavior. The fully domesticated silkworm, having lost its ability to fly, uses its wings predominantly for mating. This characteristic might explain the observed differential expression between male and female silkworms. Interestingly, increased OXPHOS protein expression in male silkworms was not detected in fruit flies. Notably, for the comparison of transcript expression between the two insects, the three most significantly enriched GO terms for highly correlated transcripts in pupae were also related to OXPHOS (Fig. 5h). Analyzing the expression dynamics of orthologs associated with these terms revealed an increase in expression levels toward later pupal stages for both species (Supplementary Fig. S15). This may indicate preparation for the higher energy demands in the transcriptome for the young adult stages in the proteome. Interestingly, expression levels decreased toward later adult stages. In all the above comparative analyses, we relied on orthologous relationships, which exclude species-specific genes.

The aim of our study was to capture broad stage-specific transcriptomic and proteomic profiles across the silkworm life cycle using a sampling strategy based on timing rather than morphology throughout development. However, this sampling strategy has limitations with regard to certain research questions. In future studies, sampling according to morphology would enable investigating molting-related processes.

Here, we present a comprehensive paired proteome and transcriptome dataset spanning key developmental stages throughout the life cycle of *B. mori*. Our dataset serves as a valuable resource for conducting comparative gene expression analyses of developmental regulatory dynamics, and the integration of publicly available data can be used to further understand developmental gene regulation across additional species.

## Methods

### Cultivation

*Bombyx mori* eggs (1O250XS) and dried mulberry leaf powder (0NP400XS) were obtained from Bombyxstore (Saint-Quentin-le-Petit, France). The eggs were incubated at room temperature and 80% humidity in an incubator for several days. Upon hatching, larvae were transferred to a new dish with freshly prepared mulberry leaf powder. The food was prepared according to the supplier’s instructions by mixing it 1:2.7 with hot water. The prepared leaf powder was stored at 4 °C. Food was replenished, and boxes were cleaned every day until cocooning (25–33 days post-hatching). Cocooning silkworms were transferred individually to 50-ml air-ventilated Falcon tubes. For mating, male and female silk moths were paired individually.

### Sample collection and processing

Whole-animal samples were collected at 17 different timepoints throughout the life cycle of *B. mori*, covering major developmental stages: 4 egg timepoints, 8 larval timepoints, 3 pupal timepoints and 2 adult conditions for males and females, each. Egg timepoints were distinguished by egg color: freshly laid (Ewhite), a few hours old (Ebrown), several days old (Eblack) and just before hatching (Eblue). Larval samples were collected at hatching (L0) and on days 3, 10, 17, 24, and 31 post-hatching (L3, L10, L17, L24 and L31, respectively), with an additional sample of larvae that climbed the walls and were ready to pupate (Lc). Pupae were collected on days 0, 3, 8 and 12 post pupation (P0, P3, P8 and P12, respectively). Adult samples included virgin females and males (AvF and AvM) and those at 5 days post mating and egg laying (AF and AM). The animals were frozen in liquid nitrogen and stored at −80 °C until further processing. For each timepoint, five replicates containing at least five animals were collected. For freshly hatched larvae, 100 individuals were pooled, and for eggs, 200 eggs were pooled per sample. For larvae on day 3 and later, the samples were first coarsely ground using a mortar and pestle in liquid nitrogen. All samples were ground in liquid nitrogen precooled containers (2-mL Eppendorf tubes) with one 7 mm steel ball (05.368.0030, Retsch) per container for 2 min at 30 Hz using a ball mill (MM 400, 20.715.0001, Retsch). Quintuplicates for each timepoint were used to measure both transcriptome and proteome from the same sample (see Supplementary Fig. S1 for a comprehensive workflow description).

### RNA extraction and NGS

For RNA extraction, 1–50 mg of cryo-beadmilled material was resuspended in 600 µl of RLT buffer with beta-mercaptoethanol. The extraction was performed according to the manufacturer’s instructions (RNeasy Mini Kit, Qiagen). An additional on-column DNase digestion was carried out using 10 µl DNase (Qiagen) and 70 µl RDD DNase buffer at RT for 15 min. The RNA pellet was resuspended in 30 µl of RNase-free water and stored at −20 °C.

NGS library preparation was performed with Lexogen’s QuantSeq 3’mRNA-Seq Library Prep Kit FWD following Lexogen’s standard protocol (015UG009V0252). Libraries were prepared with a total RNA starting amount of 22 ng and amplified in 18 PCR cycles. Libraries were profiled in high-sensitivity DNA on a 2100 Bioanalyzer (Agilent Technologies) and quantified using the Qubit dsDNA HS Assay Kit in a Qubit 2.0 Fluorometer (Life Technologies). All 96 libraries were pooled together in equimolar ratios and sequenced on one NextSeq500 high-output flow cell in single-end mode (SR) for 1 × 84 cycles plus seven cycles for the index read.

### RNA-seq data analysis

RNA-seq data included 4.6 M sequenced reads per sample on average. Analysis of the raw RNA-seq data was performed using the RNA-seq pipeline developed by the bioinformatics core facility of the Institute of Molecular Biology (IMB, available at https://gitlab.rlp.net/imbforge/NGSpipe2go). In brief, the general quality of raw reads was assessed (FastQC, 0.11.8), the reads were screened for potential contaminants (FastqScreen, 0.13), and adapters were trimmed (cutadapt, 1.18). Reads were mapped to the merged genomes of *B. mori*^102^ (GCA_014905235.2; https://silkbase.ab.a.u-tokyo.ac.jp/pub/Bomo_genome_assembly.fa.gz) and *M. notabilis* genome assemblies (https://biodb.org/tmp/morusdb/downloads/morus_notabilis_C.K.Schneid.genome.fasta.zip) using STAR 2.7.3a^103^ to exclude reads mapped to the food source. Subread (1.6) was employed for read counting, utilizing “Bomo_gene_models.gtf” (converted from https://silkbase.ab.a.u-tokyo.ac.jp/pub/Bomo_gene_models.gff3.gz) to associate reads with transcript annotations. As 3’-end sequencing was performed, the default RPKM calculation was replaced with CPM using DESeq2 (1.36.0) in R (3.6.0). The following additional tools were used: BamQC (0.1.25_devel), BamUtil (1.0.13), BEDTools (2.27), Bowtie (1.2.2), Bowtie2 (2.3.4), BWA (0.7.15), deepTools (3.1), FASTX Toolkit (0.0.14), GATK (3.4-46), HTSeq (0.6.1), Java (1.8), KentUtils (v365), MultiQC (1.7), PEAR (0.9.11), Picard (2.20), Qualimap (2.2.1), RepEnrich (1.2), rMATS (4.0.2), RSeQC (3.0.0), SAMtools (1.9), seqtk (1.3), STAR-Fusion (0.8.0), StringTie (1.3.5), TrimGalore (0.5.0), and UMI-tools (1.0.0). All data analysis tools and their respective references are listed in the Supplementary Data S16.

### *Drosophila melanogaster* data processing

We retrieved raw RNA-seq data of *D. melanogaster* from previously published data^4^ via the Sequence Read Archive (SRA). We selected specific timepoints that aligned with those of the fruit fly proteome data^12^ (Supplementary Data S11). As the number of replicates per timepoint in the database varies substantially for each timepoint, the respective Read 1 and Read 2 fastq files were concatenated separately. RNA-seq analysis was performed with the same pipeline used for *B. mori* (see the chapter above). Reads were mapped to the *D. melanogaster* genome assembly (GCA_000001215.4; https://ftp.ensembl.org/pub/release-81/fasta/drosophila_melanogaster/dna/Drosophila_melanogaster.BDGP6.dna.toplevel.fa.gz) and associated with the corresponding transcript annotation https://ftp.ensembl.org/pub/release-81/gtf/drosophila_melanogaster/Drosophila_melanogaster.BDGP6.81.gtf.gz coherent with the annotation version used for *D. melanogaster* proteome data processing^12^. The raw read counts were transformed to fragments per kilobase million mapped reads (FPKM) units using the DESeq2 (1.36.0) R package.

### Mass spectrometry sample preparation

A spoon tip of the cryo-milled insect powder was resuspended in 500 µl 1× LDS buffer (Thermo) containing 100 mM dithiothreitol (DTT), denatured for 10 min at 80 °C and sonicated for 10 min in a sonication bath (Branson) at RT. Samples were separated on a 4–12% NuPAGE NOVEX Bis-Tris gel (Thermo), with 10 µl per sample and 25 µl per egg sample at 180 V for 10 min in 1× MES buffer (Thermo Fisher Scientific). The gel was then fixed with a solution of 7% acetic acid and 40% methanol for 10 min, stained with Coomassie Brilliant Blue G250 (Sigma-Aldrich) for 10 min and destained in water overnight. For in-gel digestion, gel pieces were excised, destained with 50% EtOH/50 mM ammonium bicarbonate (ABC, Sigma) and dehydrated with acetonitrile (ACN, VWR). The gel pieces were then incubated in 10 mM DTT/50 mM ABC at 56 °C for 1 h, alkylated using 50 mM iodoacetamide/50 mM ABC (Sigma), and dehydrated with ACN, followed by overnight digestion with 1 µg trypsin (Sigma) in 50 mM ABC (Sigma)^104^. The digested peptides were desalted and stored on C18-StageTips^105^ at 4 °C until mass spectrometry (MS) measurement.

### Mass spectrometry measurement

Peptides were eluted from StageTip using 80% acetonitrile with 0.1% formic acid (solvent B). Acetonitrile was evaporated in a concentrator (Eppendorf), and the peptides were subsequently loaded onto a 50 cm column (New Objective) packed in-house with ReproSil-Pur 120 C18-AQ (particle size: 1.9 µm, Dr. Maisch GmbH) using an EasyLC 1200 system (Thermo) for peptide loading. The column, maintained at 55 °C, was mounted to the orifice of an Orbitrap Exploris 480 mass spectrometer (Thermo), and a spray voltage of 2.2 kV was applied to ionize the peptides. The instrument was operated in positive ion mode. Peptides were eluted over a 103-min optimized gradient from 3 to 40% solvent B at a flow of 250 nl min^−1^. Full scans in the mass spectrometer were conducted at a resolution of 60,000 (scan range: 300–1650 *m/z*; maximum ion trap (IT) time: 28 ms). The mass spectrometer was operated using a top 20 data-dependent acquisition mode with a minimum IT threshold of 1*10^5^ for parent ions with charge states of 2-6. A normalized collision energy of 30 and an isolation window of 1.4 were used for fragmentation, with a resolution of 15,000 per MS/MS scan.

### Mass spectrometry data analysis

MS raw files were processed with MaxQuant version 1.6.10.43^106^ using the built-in Andromeda search engine and fasta files downloaded from SilkBase^79^ (16,880 entries, silkbase.ab.a.u-tokyo.ac.jp/pub/Bomo_gene_models_prot.fa.gz) and MorusDB (26,965 entries, https://biodb.org/tmp/morusdb/downloads/morus_notabilis_C.K._Schneid.protein.fasta.zip). Standard settings were applied, “match between runs” was deactivated, and LFQ quantitation without fastLFQ activated. LFQ quantitation was restricted to unique peptides. To impute missing values, a beta distribution, derived from LFQ intensity values of all samples, was shifted to the limit of quantitation. Known contaminants, reverse database hits and protein groups only identified by site were removed. Only protein groups identified with two peptides (with one of them unique) were used for further analysis. In an additional filtering step, protein groups associated with mulberry, the *B. mori* food source, were removed. “Protein groups” represent a set of proteins that are indistinguishable because of their identification by shared peptides. Subsequent data analysis was performed using the primary protein ID of each protein group.

### Analysis of the *B. mori* developmental proteome

Imputed LFQ intensities were log_2_-transformed. To assess the correlation among replicates and across timepoints, Pearson correlation coefficients were computed for all replicates across every timepoint. The core proteome was determined using non-imputed log_2_-transformed LFQ intensities. To depict the expression of individual proteins, the mean of non-imputed log_2_-transformed LFQ intensities was utilized. Proteins without any measurement at a given timepoint were assigned to the limit of detection (LOD), which was set to the smallest measured value with a log_2_ LFQ intensity of 22.5.

The Gini ratio^107,108^ (ranging from 0 to 1) of the normalized mean difference in LFQ intensities was calculated for each combination of two timepoints for each protein. A lower score signifies stable expression, while proteins with high variability have higher scores.

$$G=\frac{{\sum }_{i=1}^{n}{\sum }_{j=1}^{n}|{x}_{i}-{x}_{j}|}{2{n}^{2}\mu }\,$$

In this equation, *n* refers to the number of timepoints, x_i_ refers to the protein quantification (LFQ intensities) at timepoint *i*, and *μ* refers to the average protein quantification across time. To identify proteins with stage-specific expression dynamics, we used self-organizing map (SOM) clustering of proteins identified in more than three replicates at any timepoint. Mean raw LFQ intensities within the upper 90th percentile of protein-specific variability (measured by IQR across timepoints) were z-scored across all timepoints. Clusters were generated using the self-organizing map (som) algorithm (“som” function^109,110^) from the kohonen (3.0.11) R package using 1000 iterations to capture complex patterns. The proteins were grouped into 16 clusters. These initial clusters were refined to 12 clusters by filtering for low mean distances to the cluster center, restricting the selection to distances falling below the 75th percentile of all intracluster distances, thereby emphasizing similarities within and differences between clusters. For the stage-specific analysis, only clusters exhibiting stage-specific expression dynamics were selected.

Fisher’s exact tests were conducted for each stage and their clusters to assess the enrichment of GO terms or Pfam domains among the proteins. The background set comprised all proteins utilized to generate the som. To control false discovery rates, the Benjamini–Hochberg procedure was applied to the *P* values.

### Orthology, functional annotation and enrichment analysis

SonicParanoid^71^ version 1.3.8 was applied to the protein sequences from SilkBase^79^ (silkbase.ab.a.u-tokyo.ac.jp/pub/Bomo_gene_models_prot.fa.gz) and FlyBase database (version r6.43, http://ftp.flybase.net/releases/FB2021_06/dmel_r6.43/fasta/dmel-all-translation-r6.43.fasta) with the parameter “run mode” set to “most-sensitive” to establish orthologous relationships between both species. The DAVID^111,112^ (Database for Annotation, Visualization and Integrated Discovery) functional annotation tool (release 2021; https://david.ncifcrf.gov) was used to retrieve the relevant Gene Ontology^113,114^ (GO), KEGG^115^ terms (Kyoto Encyclopedia of Genes and Genomes) and protein families (Pfam^116^). Pfam domains were predicted from *B. mori* protein sequences using the InterProScan^117^ tool (version 5.60-92.0, Pfam version 35.0). These were used for analyses, including only *B. mori*. To functionally characterize different gene sets, we used the retrieved functional annotations and conducted Fisher’s exact tests. To control false discovery rates, the Benjamini–Hochberg procedure was applied to the *P* values. The visualization of the functional annotations for the GO terms included only biological processes, but all GO terms are included in the supplementary data.

### Analysis of the *B. mori* developmental transcriptome

Only CPM values greater than 0 in at least one replicate at any timepoint were included. The expression values of the most variable transcripts within the upper 30th percentile of transcript-specific variability (measured by interquartile range (IQR) across timepoints) were transformed using the following formula: log_2_(CPM + 1). The Pearson correlation coefficient was computed for all replicates across every timepoint. To identify transcripts with stage-specific expression dynamics, we used unsupervised clustering of transcripts with CPM values greater than 10 in at least three samples. To focus on the dynamics of expressed transcripts, mean raw CPM values within the upper 90th percentile of transcript-specific variability (measured by IQR across timepoints) were z-scored across all timepoints. Clusters were generated using the self-organizing map (som) algorithm (‘som’ function^109,110^) from the Kohonen (3.0.11) R package with 1000 iterations, and the transcripts were grouped into 16 clusters. These initial clusters were refined to 13 clusters with mean distances below the 75th percentile of intracluster distances, thereby emphasizing similarities within and differences between clusters. For the stage-specific analysis, only clusters exhibiting stage-specific expression dynamics were selected (10 out of 13 clusters).

### Comparative analysis in Ewhite and Ebrown in relation to maternal gene expression

For the transcriptome data, we first removed rows without transcript measurements in either Ewhite or Ebrown samples. All CPM values were then log2-transformed after adding 1 to each value (log2(CPM + 1)). We included genes in the analysis only if they had CPM values ≥ 1 in at least four replicates of either Ewhite or Ebrown samples. For differential expression analysis, we performed a two-sided unpaired *t* test to compare expression levels between the two egg timepoints. The Benjamini–Hochberg procedure was applied to adjust *P* values for multiple comparisons. Transcripts were considered significantly more abundant in one timepoint if they had an adjusted *P* value ≤0.05 and had at least twice the amount in the respective timepoint. To assess functional enrichment, we conducted one-tailed Fisher’s exact tests for GO and KEGG term enrichment among the significantly abundant transcripts in Ewhite or Ebrown (as described earlier). For maternal gene enrichment analysis, we used a previously established dataset of 1534 maternal genes in *D. melanogaster*^58^ as a reference. We identified orthologous genes between *D. melanogaster* and *B. mori* (as described earlier) and removed *D. melanogaster* maternal genes with multiple *B. mori* orthologs from the dataset to avoid potential overestimation. A one-tailed Fisher’s exact test was then performed to assess the enrichment of maternal genes among the significantly abundant transcripts for Ewhite or Ebrown, respectively. The same analysis was performed with the proteome data, with the exception that differential abundance *P* values were not adjusted due to the considerably lower sensitivity of the proteomics quantification method.

### Comparative analysis of *B. mori* developmental transcriptome and proteome

The overlap between the stage-specific protein and transcript clusters was assessed for significance using Fisher’s exact test. Transcriptome and proteome datasets were log_2_-transformed (transcriptome: log_2_(CPM + 1), proteome: log_2_(LFQ)) and merged based on gene IDs. This merged dataset was used for all subsequent comparative transcriptome–proteome analyses.

Pearson's correlation coefficient was computed for all possible pairs of timepoints. We also determined gene-wise correlation between transcriptome and proteome data across the moth life cycle. For this, only proteins identified in more than three replicates at any timepoint were considered. To focus on proteins with dynamic expression, only those within the upper 90th percentile of protein-specific variability (measured by IQR across timepoints) were included. Transcriptomic data were filtered for transcripts with expression levels exceeding 10 CPM in at least three samples. Pearson correlation between the transcript and protein expression at corresponding timepoints was calculated.

Only proteins identified in more than three replicates at any timepoint and within the upper 90th percentile of protein-specific variability were included in unsupervised clustering. Transcripts exceeding 10 CPM in at least three samples and within the upper 90th percentile of transcript-specific variability (measured by IQR across timepoints) were considered. After z-scoring both datasets separately, they were merged based on shared gene IDs. Clustering was performed using the som^109,110^ function from the kohonen (3.0.11) R package with 1000 iterations, resulting in 20 clusters, which were subsequently refined to 15 clusters with mean distances below the 75th percentile of all intracluster distances. To assess stage-wise differences in transcript and protein expression within these 15 clusters, we calculated the gene-wise mean difference between both expression levels. This was done by computing the pairwise difference for each timepoint and then averaging these differences per stage. The calculated difference in the stage-wise means is referred to as the transcript–protein index. K-means clustering was used to categorize the clusters into four groups based on the similarity of their transcript–protein indexes.

### Analysis of tissue-specific transcriptome data from SilkDB in *B. mori*

Tissue-specific transcriptome expression data at different developmental stages were obtained from SilkDB^88^. To analyze genes associated with the oxidative phosphorylation (OXPHOS) pathway, SonicParanoid was used to identify orthologous relationships between protein sequences from SilkDB (*B. mori*, https://silkdb.bioinfotoolkits.net/__resource/Bombyx_mori/download/protein.fa.tar.gz) and FlyBase (*D. melanogaster*, version r6.43, http://ftp.flybase.net/releases/FB2021_06/dmel_r6.43/fasta/dmel-all-translation-r6.43.fasta), using the same parameters as previously described in the section before. OXPHOS-associated genes were then selected based on the KEGG pathway dme00190 and the established orthologous relationships between *B. mori* and *D. melanogaster*.

### Comparative analysis with *D. melanogaster*

To conduct a comparative analysis of *B. mori* and *D. melanogaster* protein data, we used previously published data covering the whole life cycle of *D. melanogaster*^12^ with similar timepoints and proteome coverage as *B. mori*. To merge the two datasets, orthologous relationships between protein sequences were established, and only proteins identified in both datasets were retained. The LFQ intensities of both datasets were then log_2_-transformed. Proteins expressed within the upper 70^th^ percentile of protein-specific variability (measured by IQR across timepoints) in any of the species were included in subsequent comparative analyses. To determine general correlations, Pearson's correlations were calculated for all pairs of timepoints of both species. To enable gene-wise correlation between both species, convolution was applied to adjust for differences in the number of timepoints of both datasets using the convolve function from the R stats (4.2.3) package. Larval timepoints of *B. mori* were convoluted from 7 to 4, and pupal timepoints for *D. melanogaster* were convoluted from 5 to 4 timepoints. Pearson's correlation was computed between both datasets for each ortholog. Pearson's correlation between the data of both species for individual stages was calculated separately.

To integrate the developmental transcriptome of *D. melanogaster*^4^, orthologous relationships were established. Both datasets were log_2_-transformed (RNA-seq measure + 1). Genes within the upper 70th percentile of transcript-specific variability (measured by IQR across timepoints) in any of the species were included. Pearson correlation was calculated between the transcriptome data of both species for all pairs of timepoints. To enable gene-wise correlation between both species, convolution was applied to adjust for differences in the number of timepoints of both datasets using the convolve function from the R stats package. Larval timepoints of *B. mori* were convoluted from 7 to 4, and pupal timepoints for *D. melanogaster* were convoluted from 5 to 4 timepoints. Pearson's correlation was computed between both datasets for each ortholog. To capture stage-specific characteristics, we computed Pearson correlations between the data of both species for individual stages separately.

### Statistics and reproducibility

All statistical analyses were conducted using R version 4.2.3 unless otherwise specified. Applied statistical tests are reported in the relevant context throughout the manuscript. Whenever multiple hypothesis testing was performed, *P* values were adjusted using the Benjamini–Hochberg procedure^118^. Visualizations were generated using the ggplot2 R package. Details on sample sizes, biological replicates, and their definitions are provided in the respective “Methods” sections.

### Reporting summary

Further information on research design is available in the Nature Portfolio Reporting Summary linked to this article.

## Supplementary information

Transparent Peer Review file

Supplementary Information

Description of Additional Supplementary Files

Supplementary Data 1-17

Reporting summary

## Data Availability

Transcriptome data have been deposited in SRA under the identifier PRJNA1096142. The mass spectrometry data have been deposited in the PRIDE database under the identifier PXD051369. Metadata detailing all timepoints and their corresponding SRA and PRIDE identifiers are provided in the Supplementary Data S17. FPKM values of all transcripts in all developmental stages in both species, proteome quantitative data, enrichment analysis results, clustering analysis results, all used data analysis scripts and all source data are available on Figshare: 10.6084/m9.figshare.27242058.

## Code availability

All custom code used in this study has been deposited to Figshare and is publicly available (10.6084/m9.figshare.27242058).
