# Supplementary material for: Unraveling developmental gene regulation in holometabolous insects through comparative transcriptomics and proteomics
Source: Commun Biol. 2025 Jul 1;8:980. doi: 10.1038/s42003-025-08414-z (PMC12216547; doi:10.1038/s42003-025-08414-z)
Supplement: Supplementary file 2 — Supplementary Information [file 42003_2025_8414_MOESM2_ESM.pdf]

Unraveling developmental gene regulation in holometabolous insects through comparative transcriptomics and proteomics

Maya Wilkens, Susanne Zimbelmann, Franziska Roth, Jasmin Cartano, Sergi Sayols, Mario Dejung, Michal Levin and Falk Butter

Supplementary Material

Supplementary Figures:

Supplementary Fig. S1: Workflow of the data analysis
Supplementary Fig. S2: Source of identified proteins
Supplementary Fig. S3: Correlation coefficients between proteome samples
Supplementary Fig. S4: Temporal transcript expression profile of the gene P25
Supplementary Fig. S5: Clustered expression profiles of proteins
Supplementary Fig. S6: Overview of the oxidative phosphorylation pathway
Supplementary Fig. S7: Number of identified transcripts per sample
Supplementary Fig. S8: Correlation coefficients between transcriptome samples
Supplementary Fig. S9: Clustered expression profiles of transcripts
Supplementary Fig. S10: Overlap between stage-specific transcriptome and proteome clusters
Supplementary Fig. S11: Clustered expression profiles of transcript and protein levels
Supplementary Fig. S12: Distribution of stage-specific transcript-protein indexes
Supplementary Fig. S13: Correlation coefficients between orthologous protein and transcript expression of *B. mori* and *D. melanogaster*
Supplementary Fig. S14: Expression profiles of proteins for the most enriched GO terms in positively correlated proteins between *B. mori* and *D. melanogaster*
Supplementary Fig. S15: Expression profiles of transcripts for the most enriched GO terms in positively correlated transcripts between *B. mori* and *D. melanogaster*
Supplementary Fig. S16: Distribution of overall protein abundance for each sample
Supplementary Fig. S17: Tissue specific RNA expression profiles of OXPHOS-associated genes (SilkDB)

Supplementary Data:

Supplementary Data S1: Enrichment analysis for the core proteome in *B. mori*
Supplementary Data S2: Enrichment analysis for proteins associated with stage-specific expression in *B. mori*
Supplementary Data S3: Enrichment analysis for the proteins associated with respective clusters in *B. mori*
Supplementary Data S4: Significantly abundant transcripts in Ewhite or Ebrown in comparison to each other, with information about maternal Drosophila genes
Supplementary Data S5: Significantly abundant proteins in Ewhite or Ebrown in comparison to each other, with information about maternal Drosophila genes
Supplementary Data S6: Gene Ontology and KEGG term enrichment for transcripts significantly abundant in Ewhite or Ebrown in comparison to each other

Supplementary Data S7: Enrichment analysis for transcripts associated with stage-specific expression in *B. mori*

Supplementary Data S8: Enrichment analysis for the transcripts associated with respective clusters in *B. mori*

Supplementary Data S9: Enrichment analysis for genes associated with negative, no (zero) and positive transcript-protein correlation in *B. mori*

Supplementary Data S10: Enrichment analysis for genes associated with groups based on transcript-protein indexes in *B. mori*

Supplementary Data S11: Selected *D. melanogaster* timepoints with corresponding SRA ids and proteome timepoints

Supplementary Data S12: Enrichment analysis for orthologs associated with negative, no (zero) and positive protein correlation between *B. mori* and *D. melanogaster*

Supplementary Data S13: Enrichment analysis of highly correlated orthologs per stage between protein levels of *B. mori* and *D. melanogaster*

Supplementary Data S14: Enrichment analysis for orthologs associated with negative, no (zero) and positive transcript correlation between *B. mori* and *D. melanogaster*

Supplementary Data S15: Enrichment analysis of highly correlated orthologs per stage between transcript levels of *B. mori* and *D. melanogaster*

Supplementary Data S16: Data analysis tools and their respective versions, references and websites

Supplementary Data S17: Metadata for all *Bombyx mori* timepoints with corresponding SRA ids, PRIDE ids and for both respective filenames

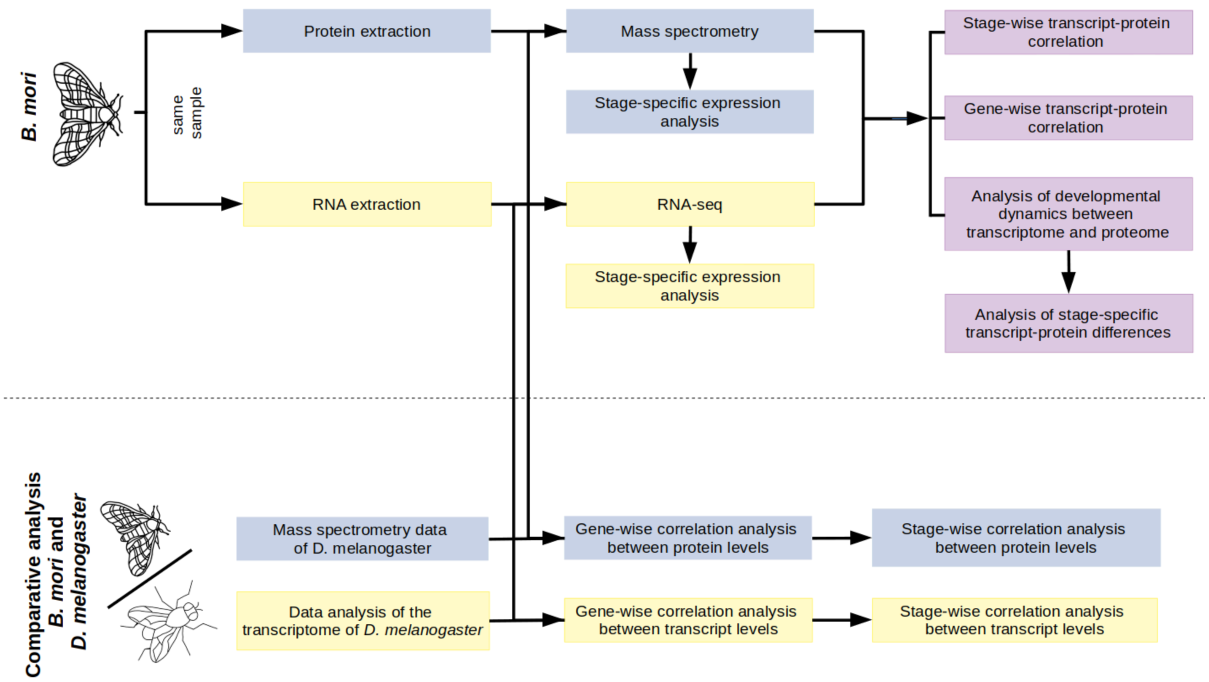

Supplementary Figure S1: Workflow of data analysis, from sample processing to stage-specific analysis, covering the transcriptome, proteome, transcriptome-proteome comparisons, and the comparative analysis between *Bombyx mori* and *Drosophila melanogaster*. The color coding represents different data types (RNA [yellow], protein [blue] and RNA-protein comparative analyses [purple]), and is consistently used throughout the manuscript. The developmental proteome and transcriptome data of *D. melanogaster* were retrieved from Casas-Vila et al., 2017 Genome Research and Graveley et al., 2011 Nature, respectively.

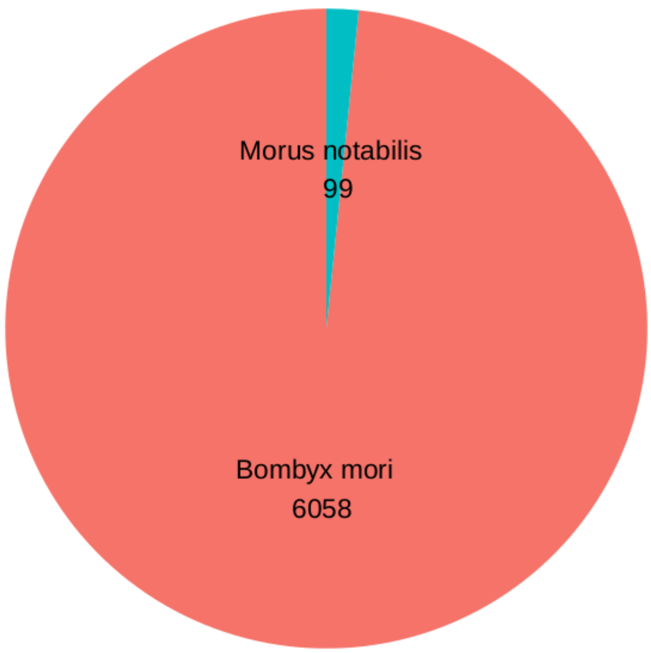

Supplementary Figure S2: Pie chart of identified protein groups of *Bombyx mori* and the food source *Morus notabilis*. Mulberry proteins represent only 0.02 % of the overall identified proteins (6,157 proteins).

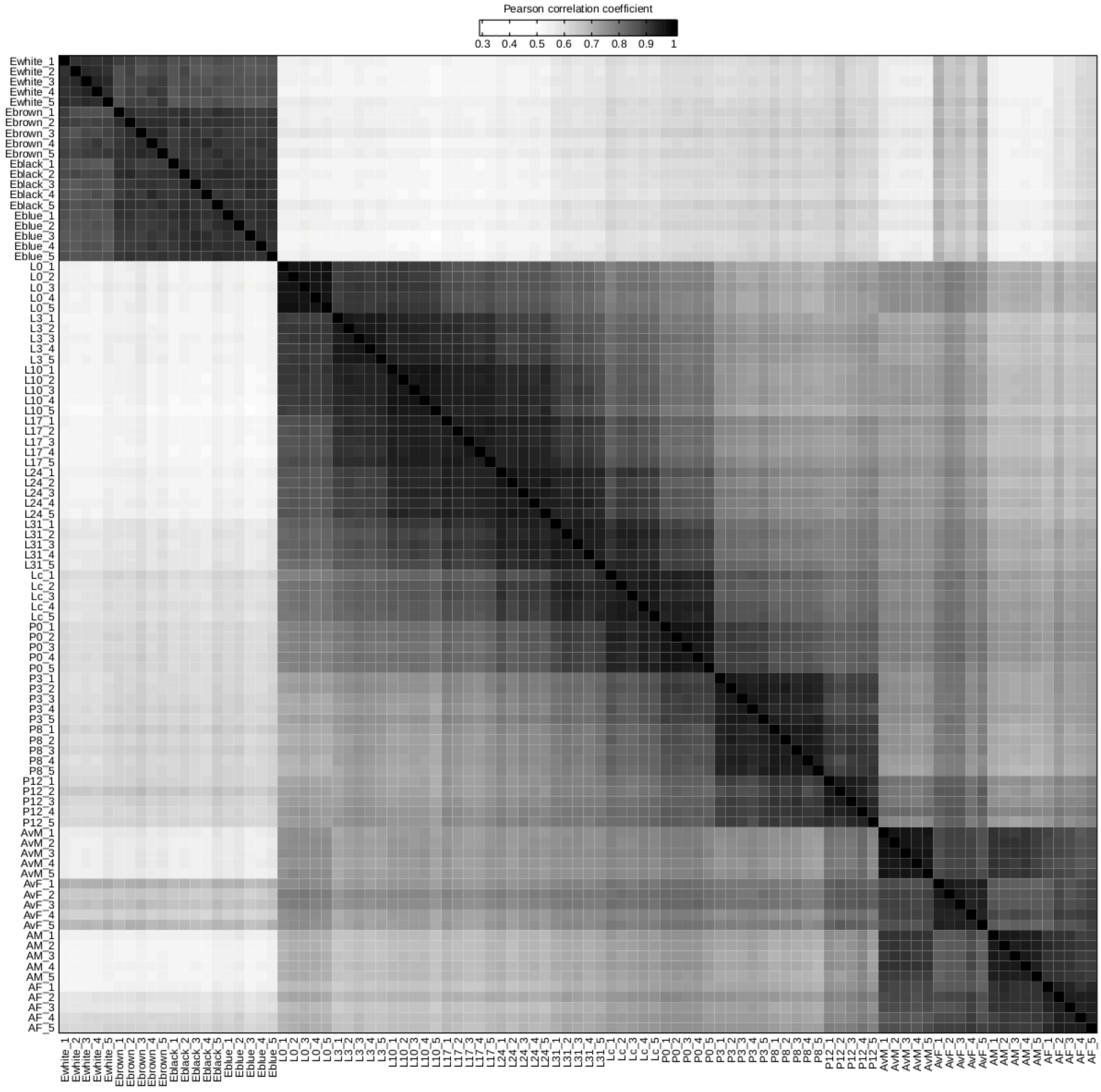

Supplementary Figure S3: Heatmaps depicting the Pearson correlation coefficients between the proteomes across the 17 timepoints covering the whole life cycle of *B. mori.* Proteome correlations were computed from log_2-transformed LFQ intensities.

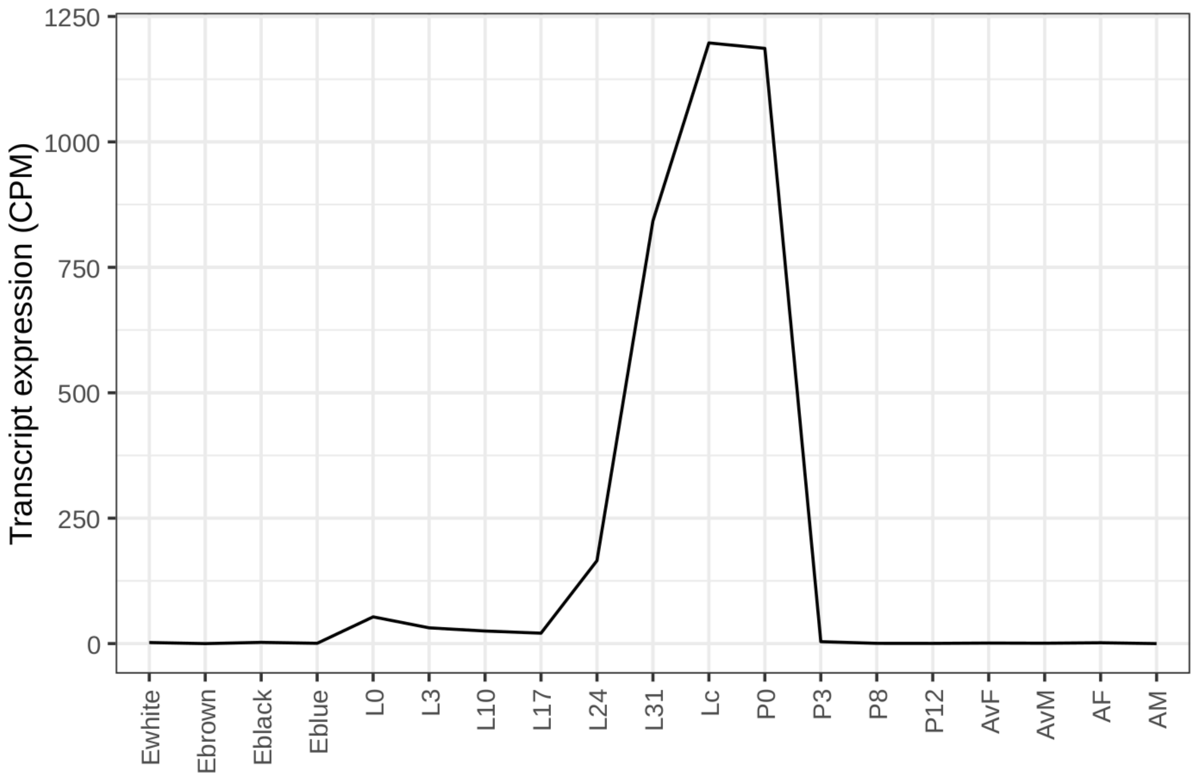

Supplementary Figure S4: Temporal transcript expression profile (mean CPM values, *n* = 5 biological replicates except for L10, L17, L24 and L31, for which *n* = 4) of the gene P25.

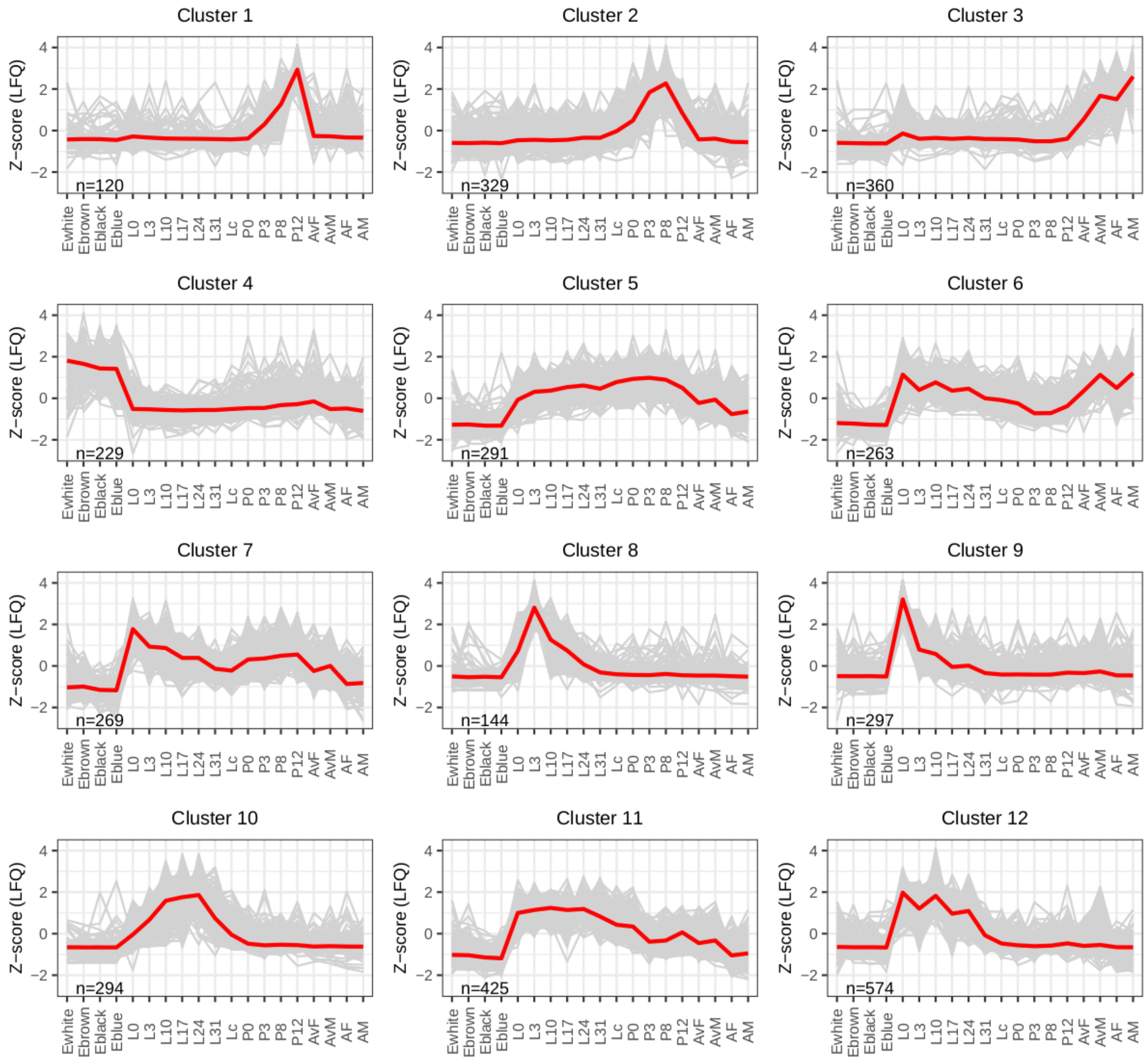

Supplementary Figure S5: Expression profiles of proteins assigned to the respective clusters (cluster-wise median z-score of mean LFQ intensities) generated by self-organizing map (SOM) clustering. Gray lines represent individual z-score normalized protein expression profiles, while the red line represents the median expression level across proteins in each cluster.

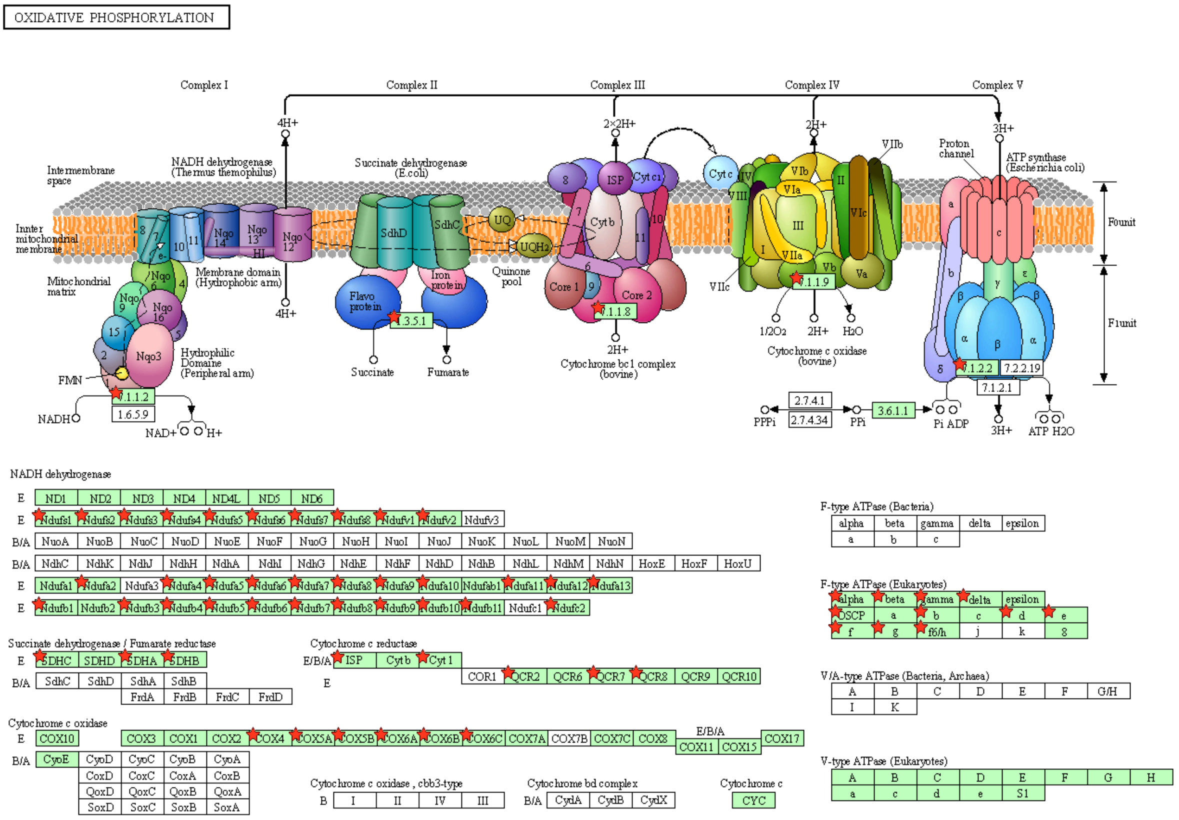

Supplementary Figure S6: Overview of the oxidative phosphorylation pathway in *Drosophila melanogaster* with orthologs of adult-specific *Bombyx mori* proteins highlighted by red stars. Green cells represent *D. melanogaster* proteins and white cells represent proteins associated with the pathway from other organisms (KEGG pathway ID: dme00190, obtained from the KEGG database).

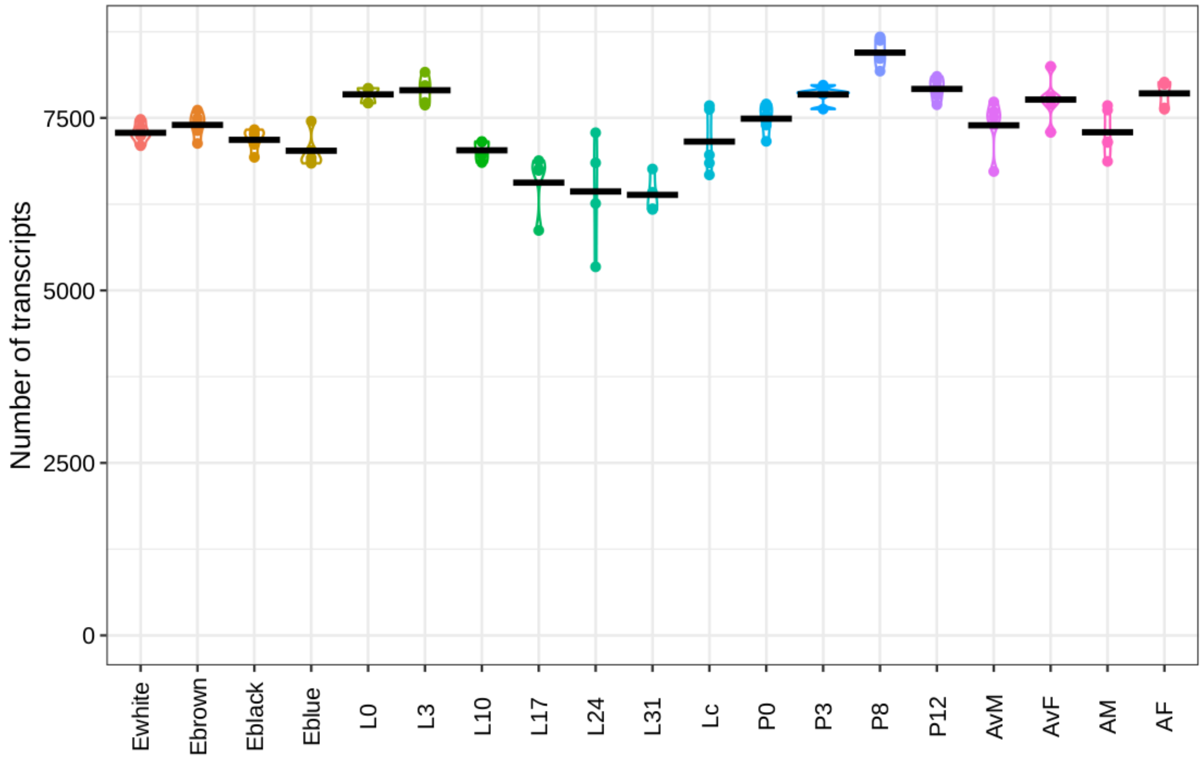

Supplementary Figure S7: Violin plot representing the distribution of the number of identified transcripts across replicates for each timepoint in the developmental life cycle of *B. mori*. The mean number of identified transcripts is indicated by the black line, while each point within the violin plot represents a replicate.

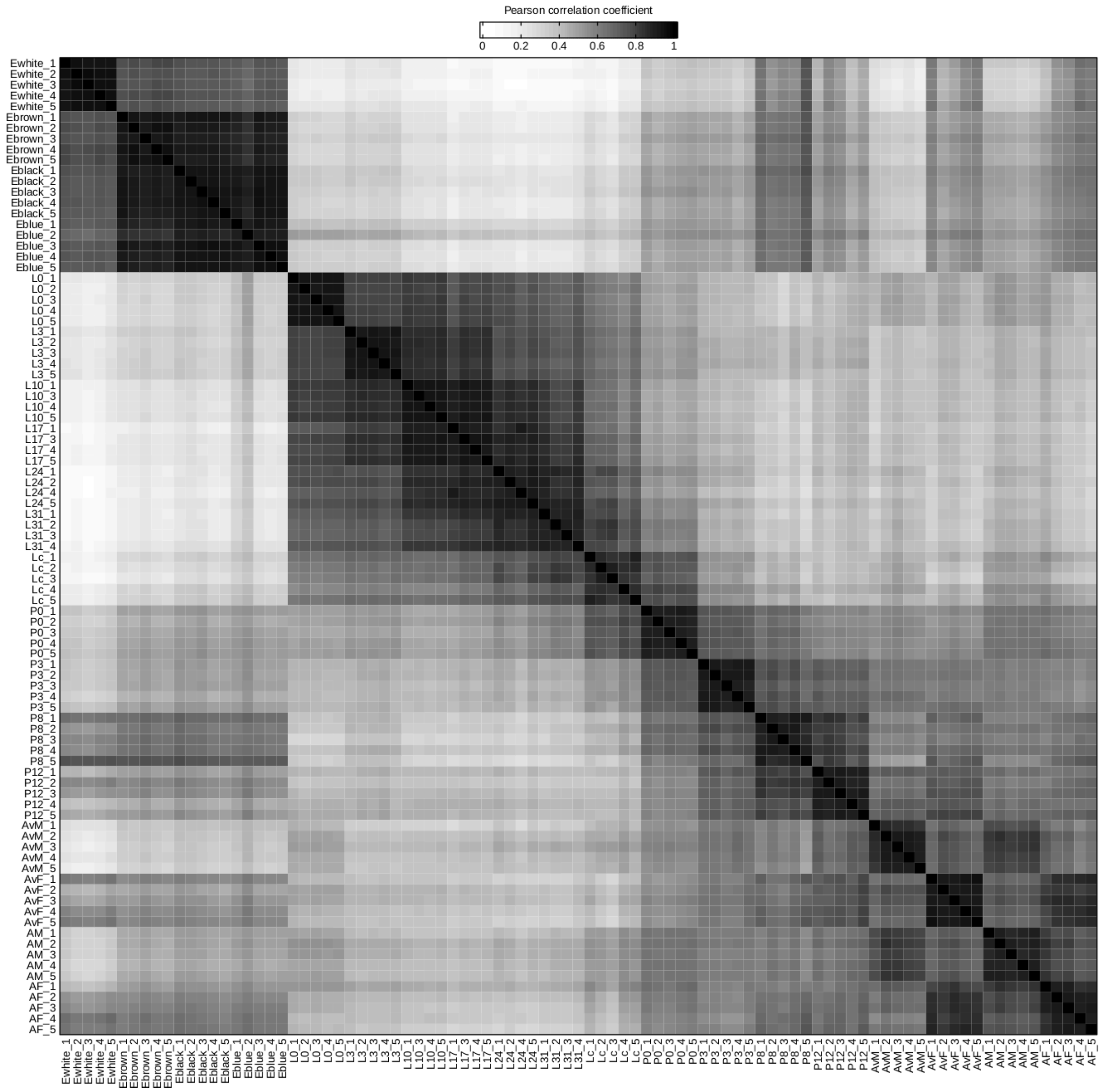

Supplementary Figure S8: Heatmaps depicting the Pearson correlation coefficients between the transcriptomes across the 17 timepoints covering the whole life cycle of *B. mori.* Transcriptome correlations were computed from the 30th percentile of the most variable transcript expression for $\log_2$(CPM + 1).

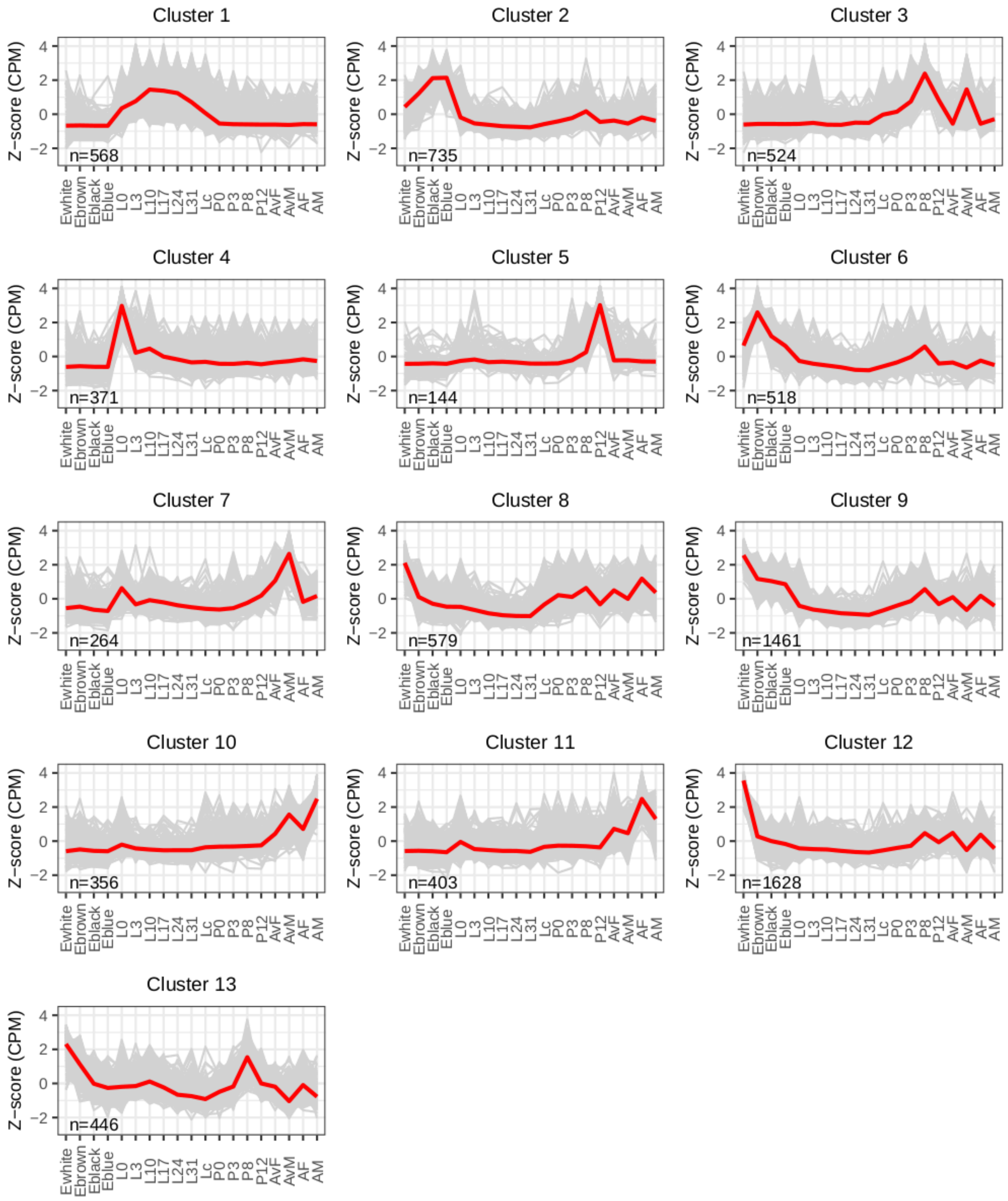

Supplementary Figure S9: Expression profiles of transcripts assigned to the respective clusters (cluster-wise median z-score of mean CPM values) generated by self-organizing map (SOM) clustering. Gray lines represent individual z-score normalized transcript expression profiles, while the red line represents the median expression level across transcripts in each cluster.

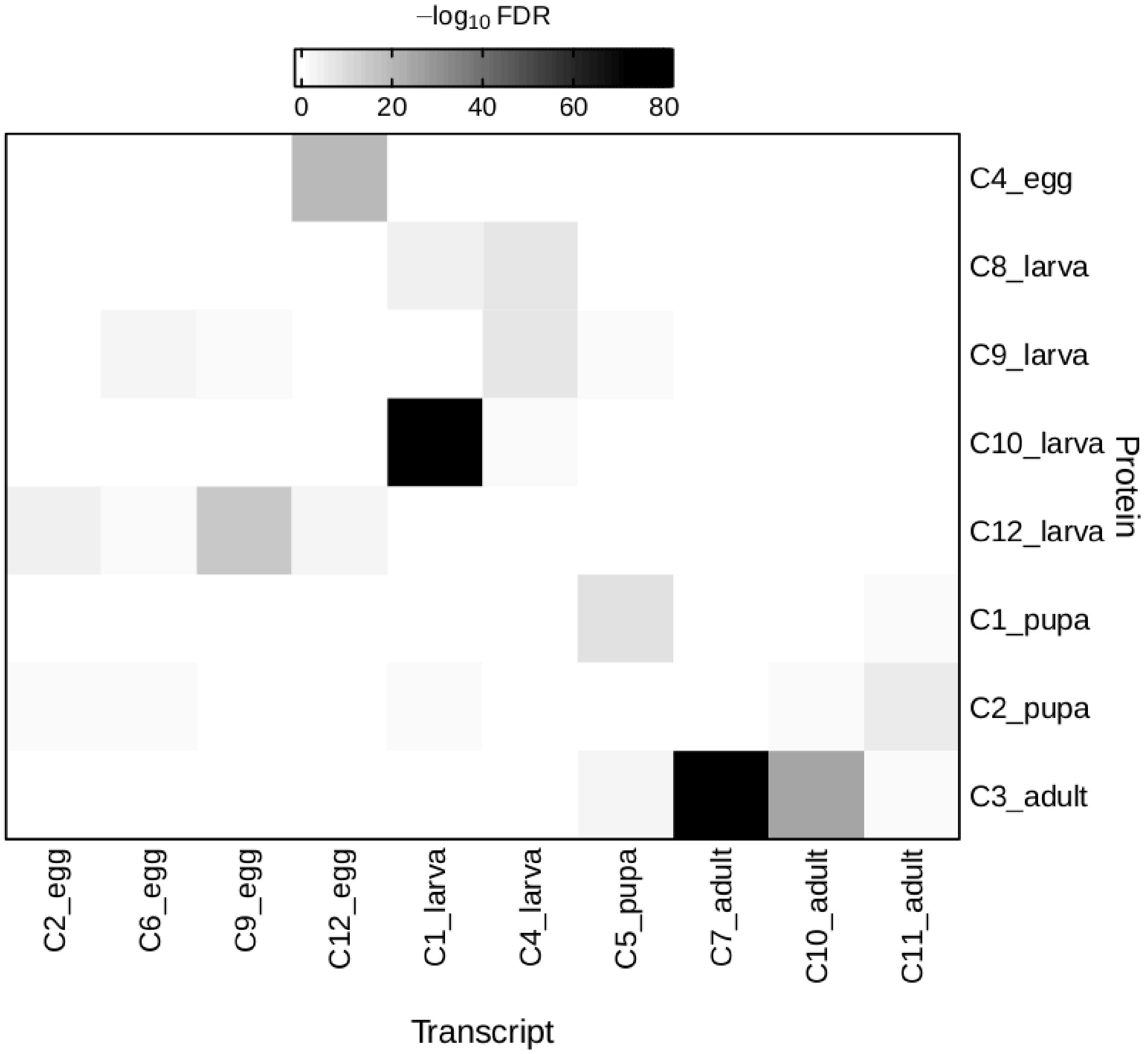

Supplementary Figure S10: Heatmap displaying the significance of overlap between the stage-specific transcriptome and proteome clusters of *B. mori*. Each coloured cell represents the adjusted p-value (FDR) between the corresponding pair of proteome and transcriptome clusters, calculated using Fisher's exact test. Only p-values for overlaps with at least 5 genes are shown.

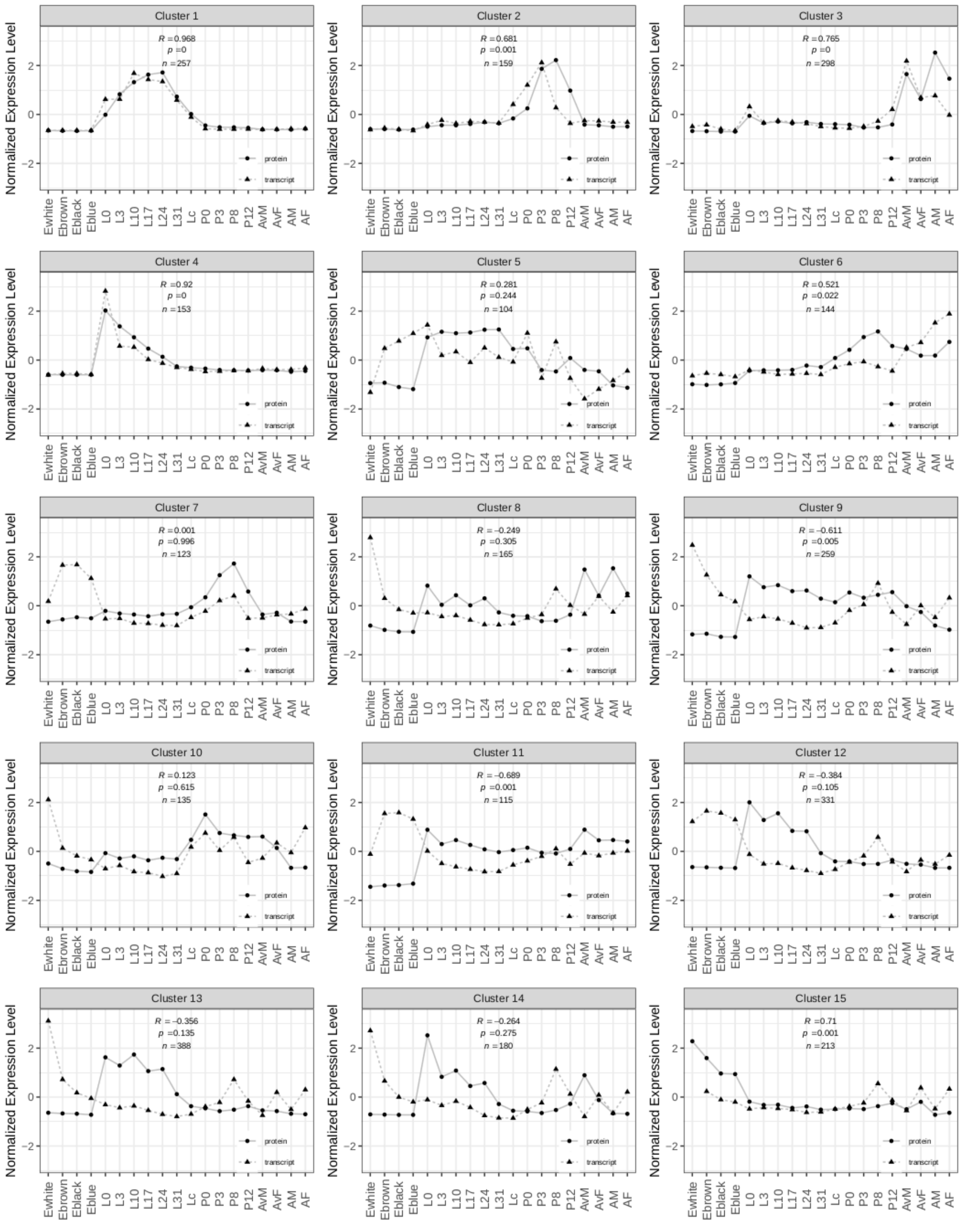

Supplementary Figure S11: Protein (dashed line) and transcript (solid line) expression levels of genes assigned to the respective clusters (cluster-wise median z-score of mean CPM values and mean LFQ values) generated by unsupervised SOM clustering. Additionally, for each cluster the Pearson correlation coefficient between protein and transcript expression levels and the corresponding p-value is depicted.

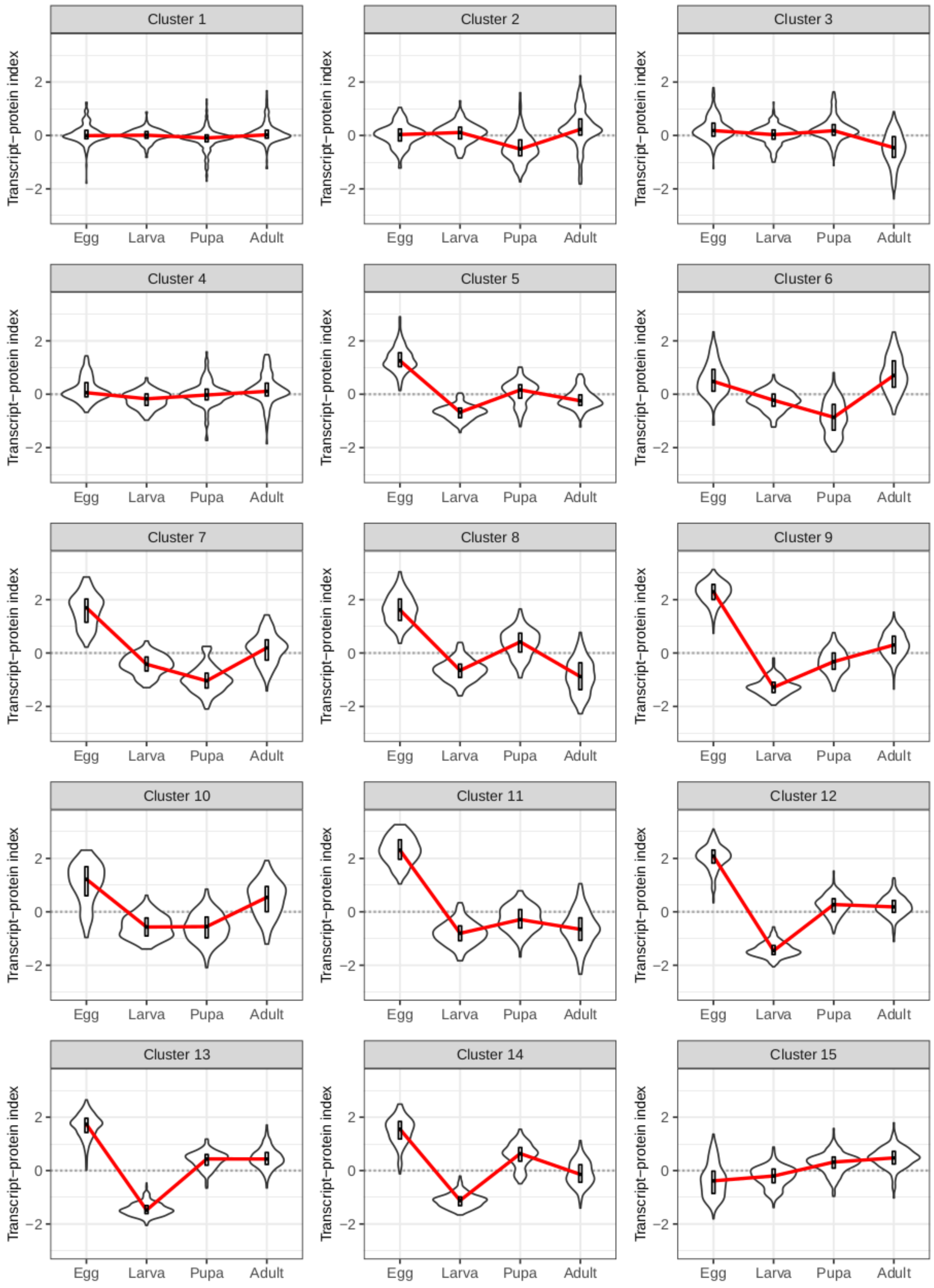

Supplementary Figure S12: Violin plots illustrate the stage-specific transcript-protein index (representing the difference in means between transcript and protein expression). The red line connects stage-specific mean transcript-protein indexes (indicated as horizontal lines in the box plots with the upper and lower edges of the box representing the interquartile range). Clusters are the same as in Supplementary Figure S9.

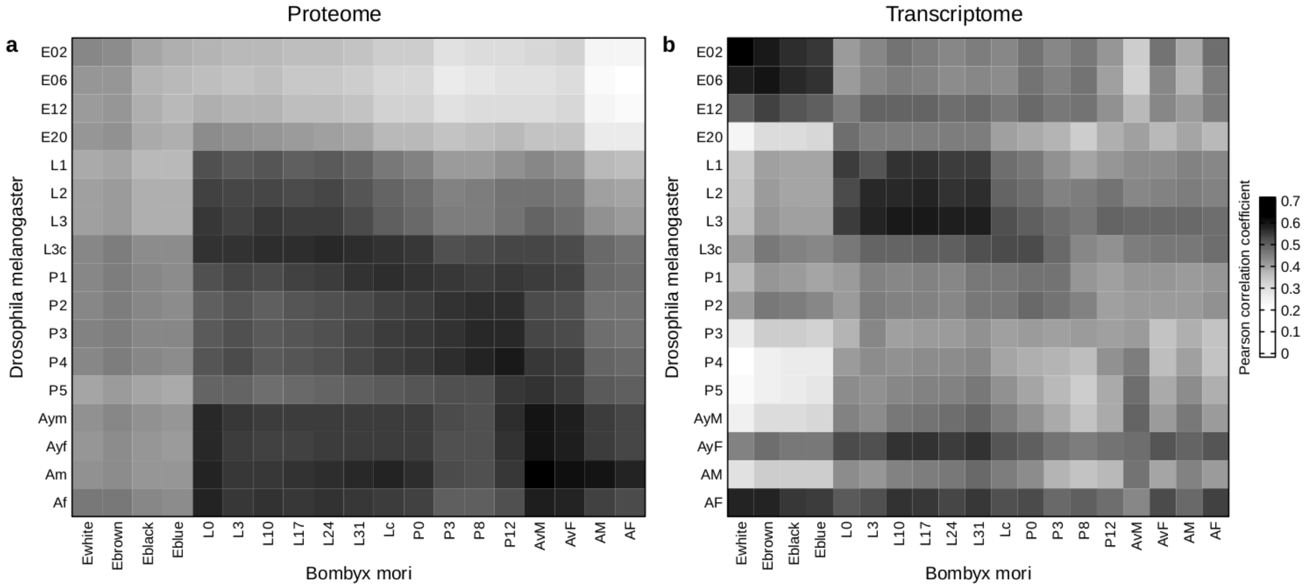

Supplementary Figure S13: Heatmaps displaying the Pearson correlation coefficient between **a** the orthologous protein expression of *B. mori* and *D. melanogaster* and between **b** the orthologous transcript expression of both insects across all timepoints (*n* = 3,412 orthologous proteins identified in both species and *n* = 5,986 orthologous transcripts identified in both species).

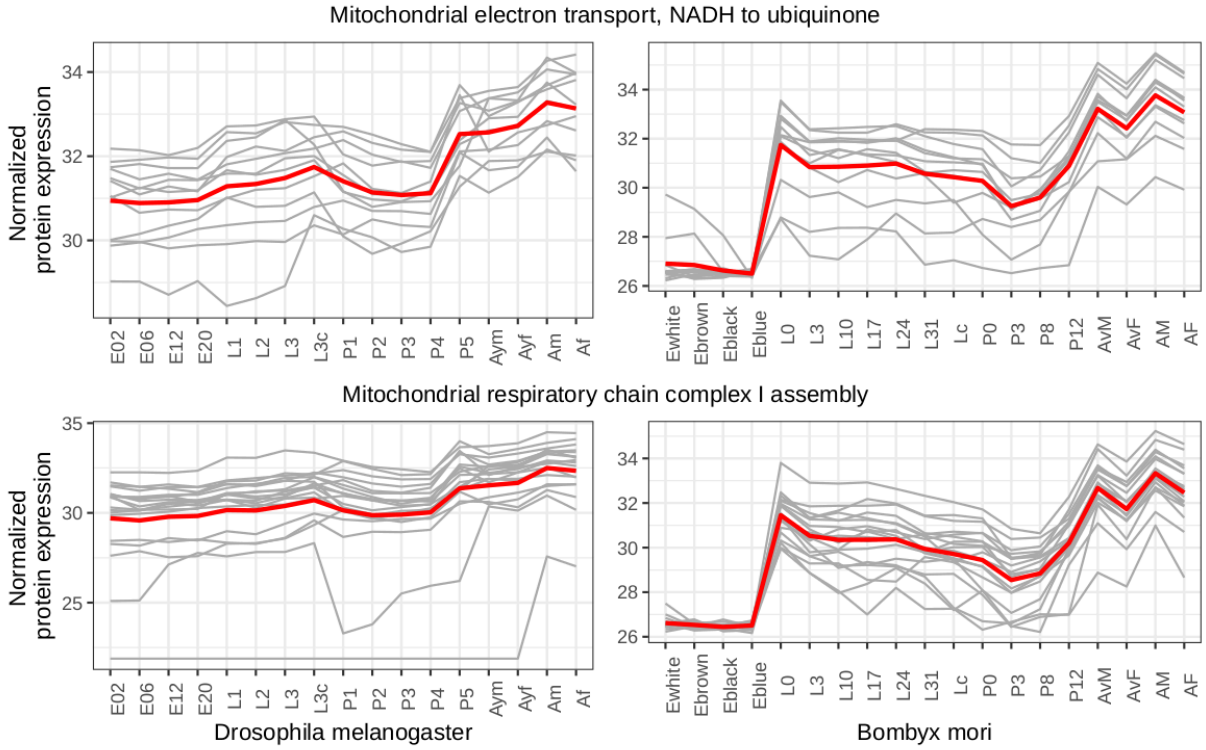

Supplementary Figure S14: The expression profiles of orthologous proteins associated with the two most significantly enriched GO terms within the significantly positively correlated proteins between *B. mori* and *D. melanogaster* are illustrated across all timepoints of the life cycles of both insects (*D. melanogaster* - left; *B. mori* - right). Gray lines represent individual protein expression profiles, while the red line represents the mean expression level across proteins in each cluster.

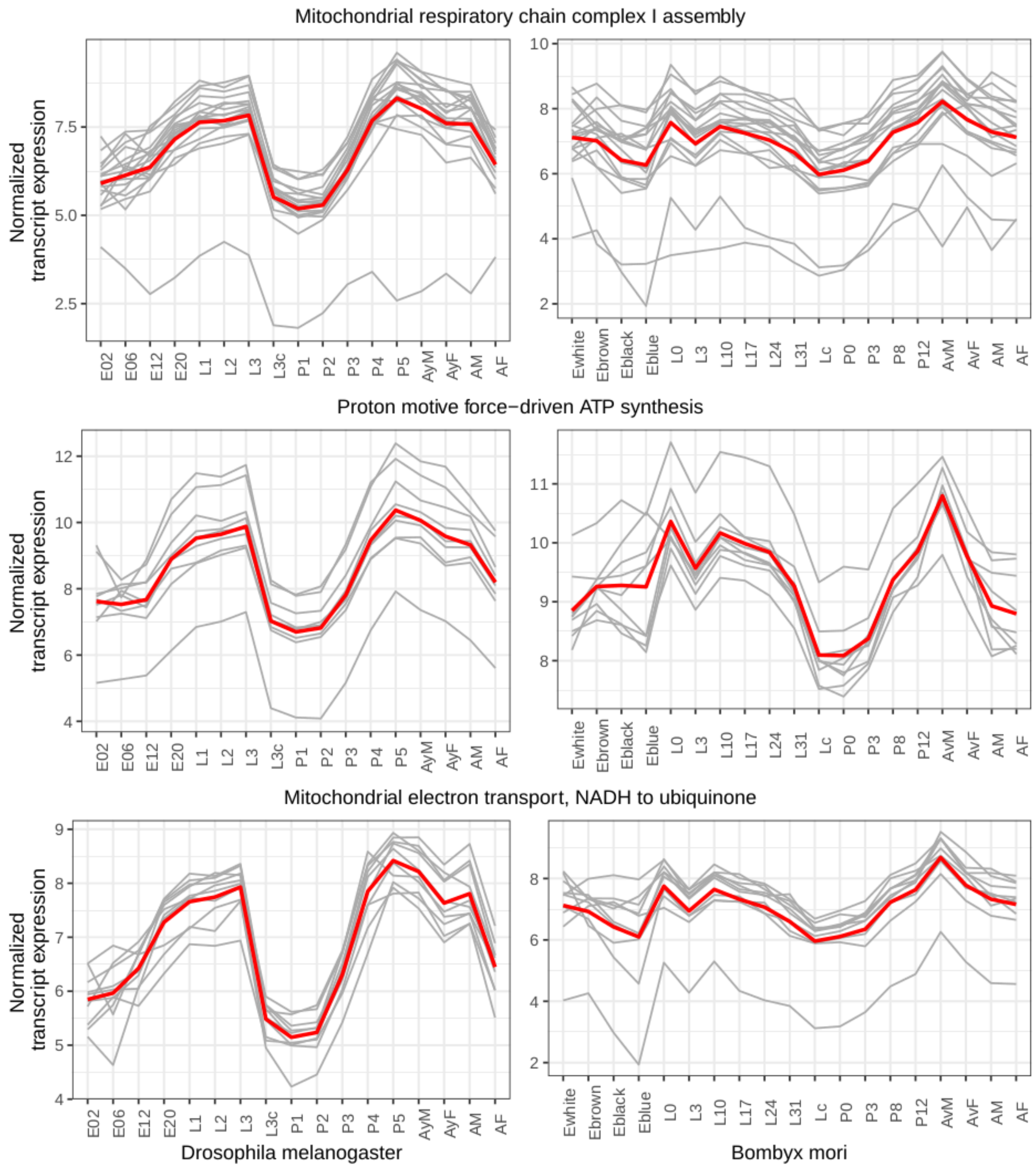

Supplementary Figure S15: The expression profiles of orthologous transcripts associated with the three most significantly enriched GO terms within the significantly positively correlated transcripts between *B. mori* and *D. melanogaste*r are illustrated across all timepoints of the life cycles of both insects (*D. melanogaste*r - left; *B. mori* - right) . Gray lines represent individual transcript expression profiles, while the red line represents the mean expression level across transcripts in each cluster.

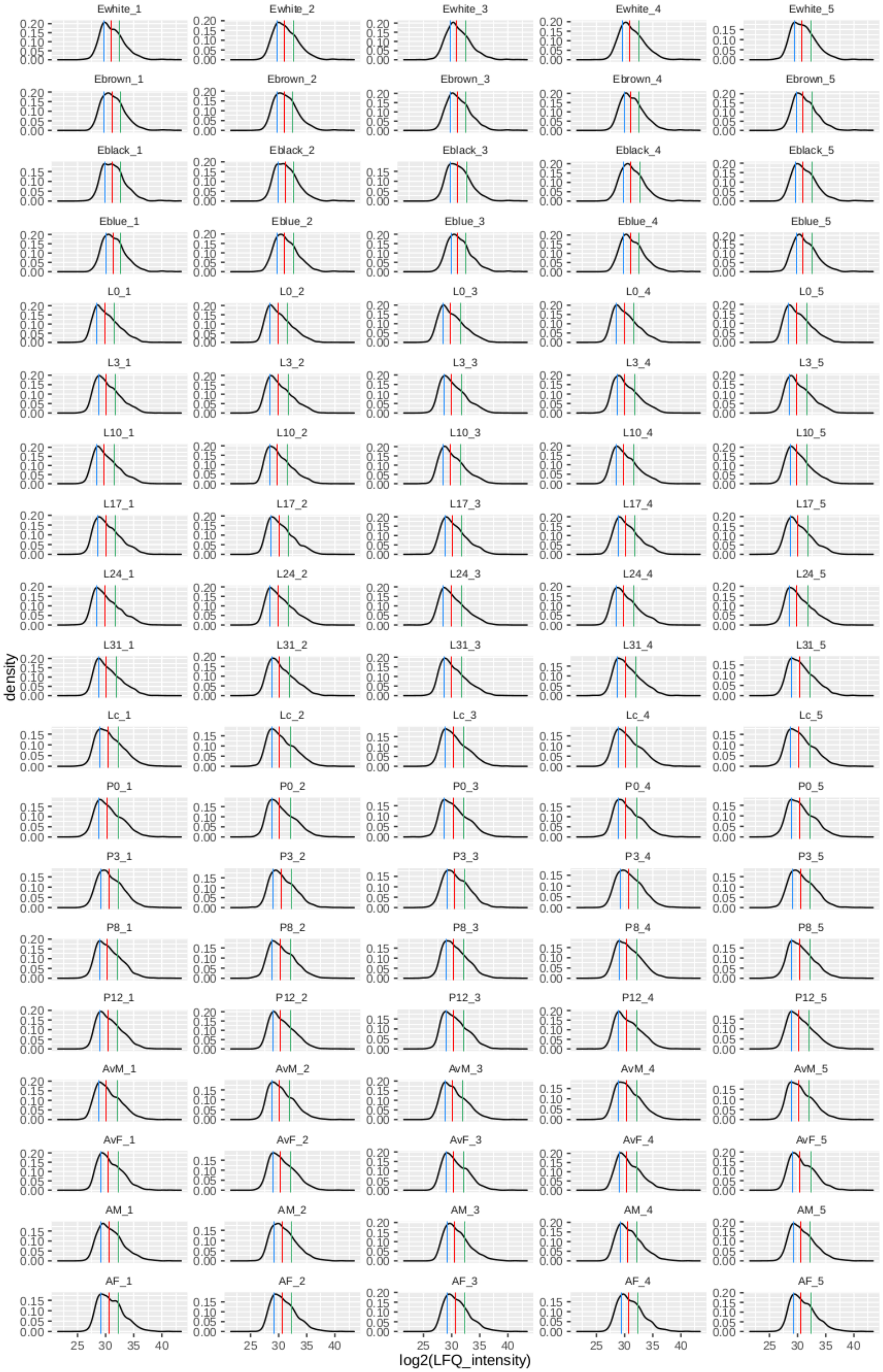
Ewhite_1
Ewhite_2
Ewhite_3
Ewhite_4
Ewhite_5
Ebrown_1
Ebrown_2
Ebrown_3
Ebrown_4
Ebrown_5
Eblack_1
Eblack_2
Eblack_3
Eblack_4
Eblack_5
Eblue_1
Eblue_2
Eblue_3
Eblue_4
Eblue_5
L0_1
L0_2
L0_3
L0_4
L0_5
L3_1
L3_2
L3_3
L3_4
L3_5
L10_1
L10_2
L10_3
L10_4
L10_5
L17_1
L17_2
L17_3
L17_4
L17_5
L24_1
L24_2
L24_3
L24_4
L24_5
L31_1
L31_2
L31_3
L31_4
L31_5
Lc_1
Lc_2
Lc_3
Lc_4
Lc_5
P0_1
P0_2
P0_3
P0_4
P0_5
P3_1
P3_2
P3_3
P3_4
P3_5
P8_1
P8_2
P8_3
P8_4
P8_5
P12_1
P12_2
P12_3
P12_4
P12_5
AvM_1
AvM_2
AvM_3
AvM_4
AvM_5
AvF_1
AvF_2
AvF_3
AvF_4
AvF_5
AM_1
AM_2
AM_3
AM_4
AM_5
AF_1
AF_2
AF_3
AF_4
AF_5
density
0.00
0.05
0.10
0.15
0.20
25
30
35
40
log2(LFQ_intensity)

Supplementary Figure S16: Density plots illustrate the distribution of overall protein levels (log_2-transformed LFQ intensities) across all timepoints for each sample. The blue lines within each plot represent the 25th percentile, the red lines the median and the green lines the 75th percentile of each sample.

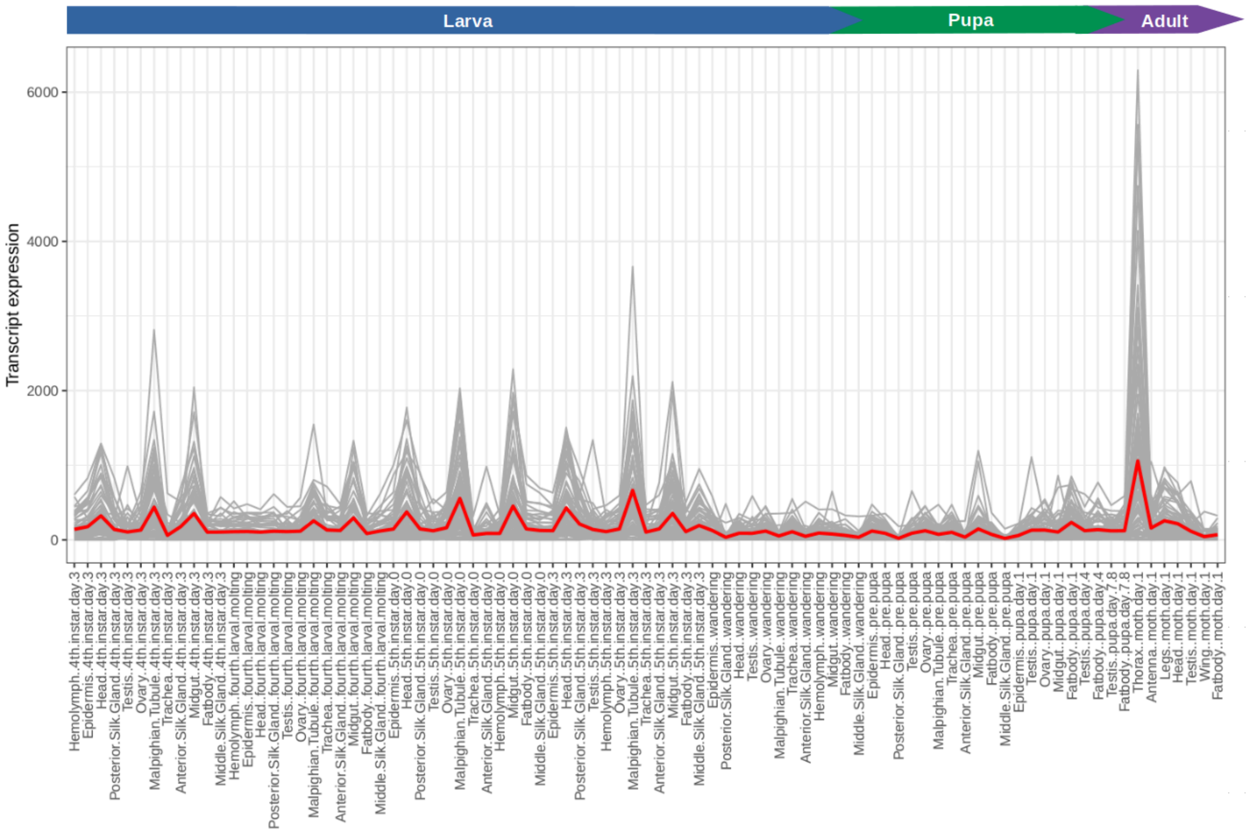

Supplementary Figure S17: RNA expression profiles of genes associated with the oxidative phosphorylation pathway (80 genes) (dme00190, obtained from the KEGG database). Data stems from previously published tissue-specific transcriptome data at different developmental stages from SilkDB (Lu et al., 2020) in *B. mori*. Gray lines represent individual transcript expression profiles, while the red line represents the mean expression level across transcripts. Thorax moth day 1 sample exhibits highest mean expression levels.
