# Supplementary material for: Unraveling developmental gene regulation in holometabolous insects through comparative transcriptomics and proteomics
Source: Commun Biol. 2025 Jul 1;8:980. doi: 10.1038/s42003-025-08414-z (PMC12216547; doi:10.1038/s42003-025-08414-z)
Supplement: Supplementary file 3 — Description of Additional Supplementary Files [file 42003_2025_8414_MOESM3_ESM.pdf]

Description of Additional Supplementary Files

Supplementary Data S1: Enrichment analysis for the core proteome in B. mori

Supplementary Data S2: Enrichment analysis for proteins associated with stage-specific expression in B. mori

Supplementary Data S3: Enrichment analysis for the proteins associated with respective clusters in B. mori

Supplementary Data S4: Significantly abundant transcripts in Ewhite or Ebrown in comparison to each other, with information about maternal Drosophila genes

Supplementary Data S5: Significantly abundant proteins in Ewhite or Ebrown in comparison to each other, with information about maternal Drosophila genes

Supplementary Data S6: Gene Ontology and KEGG term enrichment for transcripts significantly abundant in Ewhite or Ebrown in comparison to each other

Supplementary Data S7: Enrichment analysis for transcripts associated with stage specific expression in B. mori

Supplementary Data S8: Enrichment analysis for the transcripts associated with respective clusters in B. mori

Supplementary Data S9: Enrichment analysis for genes associated with negative, no (zero) and positive transcript protein correlation in B. mori

Supplementary Data S10: Enrichment analysis for genes associated with groups based on transcript-protein indexes in B. mori

Supplementary Data S11: Selected D. melanogaster timepoints with corresponding SRA ids and proteome timepoints

Supplementary Data S12: Enrichment analysis for orthologs associated with negative, no (zero) and positive protein correlation between B. mori and D. melanogaster

Supplementary Data S13: Enrichment analysis of highly correlated orthologs per stage between protein levels of B. mori and D. melanogaster

Supplementary Data S14: Enrichment analysis for orthologs associated with negative, no (zero) and positive transcript correlation between B. mori and D. melanogaster

Supplementary Data S15: Enrichment analysis of highly correlated orthologs per stage between transcript levels of B. mori and D. melanogaster

Supplementary Data S16: Data analysis tools and their respective versions, references and websites

Supplementary Data S17: Metadata for all Bombyx mori timepoints with corresponding SRA ids, PRIDE ids and for both respective filenames
