# Supplementary material for: Unraveling developmental gene regulation in holometabolous insects through comparative transcriptomics and proteomics
Source: Commun Biol. 2025 Jul 1;8:980. doi: 10.1038/s42003-025-08414-z (PMC12216547; doi:10.1038/s42003-025-08414-z)
Supplement: Supplementary file 1 — Transparent Peer Review file [file 42003_2025_8414_MOESM1_ESM.pdf]

nature portfolio

Peer Review File

Unraveling developmental gene regulation in holometabolous insects through comparative transcriptomics and proteomics

Corresponding Author: Dr Michal Levin

This file contains all reviewer reports in order by version, followed by all author rebuttals in order by version.

Version 0:

Reviewer comments:

Reviewer #1

(Remarks to the Author)
In this manuscript, the authors performed proteome and transcriptome analysis using eggs, larvae, pupae, and virgin and mated adults of B. mori. Using proteome and transcriptome data, the dynamicity of the developmental proteome and transcriptome of B. mori were revealed and, stage-specific proteins and transcripts were identified, and key roles of each stage were found. Moreover, transcript-protein correlations also were found. Then, the authors performed the comparative analysis of developmental systems using their B. mori transcriptome proteome data and D. melanogaster transcriptome and proteome data in public databases, revealing that there are similar correlations of proteins and genes and stage-specific analysis revealed that larvae had the greatest coherence in datasets of two species.
In my opinion, this manuscript is worth to publishing in communications biology, because they revealed several new and basic insights into each developmental stage. As the authors described, their provided data must be valuable resources for meta-analysis and B. mori or insect species research. However, there are some points which the authors must revise before publication.
Most important one is that the proteome and transcriptome data and its related data must be available in public data repository (such as "figshare" (URL: https://figshare.com/)) and DOIs of each data should be shown in "Data availability" part of manuscript for ensuring availability and reproducibility of data analysis. I listed data the author must upload .
1. FPKM value data of all transcripts in all developmental stages in both species
2. Calculated proteome quantitative data of B. mori
3. All output data of enrichment analysis (such as DAVID and GO analysis)
4. Output files of clustering analysis
5. Files containing the scripts of all data analysis
Moreover, meta-data of raw RNA-Seq data of B. mori must be shown as in Supplementary Data S8, and the mass spectrometry data must be uploaded in the data repository with DOI, which I showed above.

The other point is that comprehensive workflow of data analysis must be added in main figure to make the readers better understanding the article (as figure 1 of Zheng et al., (2024), URL: https://www.nature.com/articles/s41597-024-03855-3, or figure 1 of Rahuman et al., (2024), URL: https://www.nature.com/articles/s41597-024-03541-4).
In line 144, the authors described lack of GO annotation in B. mori. I suggested GO enrichment analysis using other annotation data in Bono et al., (2022) (URL: https://doi.org/10.3390/insects13070586) ().

Minor points
1. Accession IDs of genome data of D. melanogaster and B. mori must be added in ”Methods” part (lines 744-749).
2. URLs and references of all data analysis tools must be shown (lines 732-737).

Reviewer #2

(Remarks to the Author)
In this manuscript the authors collected transcriptomic and proteomic data along with the development of the silkworm from egg to adult. This is a novel a novel attempt since these data are obtained as pairs and could be of relevant scientific value. However, I have two major concerns for the publication of the manuscript.

1. The title refers to the study as systems biology and indeed it contains some systems biological analyses, but they hardly

occupy major portion of this study. I'd rather feel this paper as a bioinformatics study.
2. Reproducibility of the data
Every study that refers to development must be conducted with the extreme cares on the staging of the organism since that is the only way to describe objectively what is under analysis. Detailed description of the embryonic development based on the morphology of Bombyx mori embryo can be found in several literatures and the authors should be aware of it. One can easily describe the embryonic stages of the samples by observing embryos by dissecting small numbers of the eggs if they are well synchronized. Post-embryonic development of insect should be described in concern with the molting cycle since many biological processes are regulated in accordance with the molting cycle which is in vast part controlled by the endocrinological conditions. Therefore, in this context the authors should provide at least on which day of which instar they collected samples, not the days after hatching. Moreover, the larval duration widely varied under the rearing conditions provided, suggesting the rearing condition was suboptimal. Nevertheless, without providing standard results of breeding (e.g. duration of each instar) for the strain used, one cannot evaluate the data in terms of silkworm biology.

It looks inevitable to reevaluate all the data, and my suggestion is to concentrate on the earliest two stages which may mark the switching of maternal and zygotic gene expression and protein production.

Version 1:

Reviewer comments:

Reviewer #1

(Remarks to the Author)
The authors well addressed my comments. I deeply appreciate their efforts. I recommend the manuscript is worth the publication.

Reviewer #2

(Remarks to the Author)
Through the revision of the manuscript, authors are well aware of the comments I placed and though I found them not absolutely satisfactory but acceptable. One issue I have to emphasize is the reproducibility of the results. Since the sampling timings are not staged along the developmental process, especially during larval stage, which authors admit, it is not realistic to reproduce the results shown in this paper by other hands. In my opinion, addition of good reasoning to claim the significance of the data shown (even without staged sampling) will avoid spoiling the value of this paper.

Minor points
L54 I assume authors want to mean "80% of the species from this class (if referring Insecta) or subphylum (if referring Hexapoda)".
L56 Stages of hemimetabolous insect are egg, nymph, adult and nymph is not considered as equivalent to larva, so it is inaccurate to describe it as if it is only lacking pupal stage.
L58 "Holometabola comprises various insect orders"
Holometabola is insect so using "insect order" for describing it is apparently duplicated expression. In addition, it is not clear what kind of variation the authors want to mean by including "various". In my opinion "Holometabola comprises 11 orders," is appropriate here.
L67 "D. melanogaster and B. mori, which belong to a monophyletic group"
This is quite confusing since all the insects belong to a monophyletic group, all the holometabolous insects belong to yet another monophyletic group. I suggest including the name of this clade Mecopterida (or Panorpida). Reference 37 is not appropriate here.
L91 "crawling upward to prepare for pupation"
Proper technical term is "wandering"
L92 "pupa" -> pupae
L124 To my knowledge, it is not widely known that fibrohexamerin (formerly known as P25) gene is expressed during pupal stage, since it's believed that the expression of this gene is restricted to the posterior silk gland, and this tissue degenerates during pupal molt. I urge the authors to provide references if this expression timing has already been reported, otherwise please provide insight on why you are detecting this, and which tissue is the provisional expression site. Likewise, cocoonase gene expression in adults is also not expected. These might be minor points in the context of this study, but quite intriguing results.
L125 pupation -> pupae

Version 2:

Reviewer comments:

Reviewer #2

(Remarks to the Author)
The authors have thoroughly addressed my comments, and I sincerely appreciate their efforts. I believe the manuscript is well-suited for publication

Open Access This Peer Review File is licensed under a Creative Commons Attribution 4.0 International License, which permits use, sharing, adaptation, distribution and reproduction in any medium or format, as long as you give appropriate credit to the original author(s) and the source, provide a link to the Creative Commons license, and indicate if changes were made.
In cases where reviewers are anonymous, credit should be given to 'Anonymous Referee' and the source.
The images or other third party material in this Peer Review File are included in the article's Creative Commons license, unless indicated otherwise in a credit line to the material. If material is not included in the article's Creative Commons license and your intended use is not permitted by statutory regulation or exceeds the permitted use, you will need to obtain permission directly from the copyright holder.
To view a copy of this license, visit https://creativecommons.org/licenses/by/4.0/

Point-by-point responses to the referee's comments

<u>Reviewer #1</u> (Remarks to the Author):

In this manuscript, the authors performed proteome and transcriptome analysis using eggs, larvae, pupae, and virgin and mated adults of B. mori. Using proteome and transcriptome data, the dynamicity of the developmental proteome and transcriptome of B. mori were revealed and, stage-specific proteins and transcripts were identified, and key roles of each stage were found. Moreover, transcript-protein correlations also were found. Then, the authors performed the comparative analysis of developmental systems using their B. mori transcriptome proteome data and D. melanogaster transcriptome and proteome data in public databases, revealing that there are similar correlations of proteins and genes and stage-specific analysis revealed that larvae had the greatest coherence in datasets of two species.

In my opinion, this manuscript is worth to publishing in communications biology, because they revealed several new and basic insights into each developmental stage. As the authors described, their provided data must be valuable resources for meta-analysis and B. mori or insect species research. However, there are some points which the authors must revise before publication.

- Most important one is that the proteome and transcriptome data and its related data must be available in public data repository (such as "figshare" (URL: https://figshare.com/)) and DOIs of each data should be shown in "Data availability" part of manuscript for ensuring availability and reproducibility of data analysis. I listed data the author must upload .

  1. FPKM value data of all transcripts in all developmental stages in both species
  2. Calculated proteome quantitative data of B. mori
  3. All output data of enrichment analysis (such as DAVID and GO analysis)
  4. Output files of clustering analysis
  5. Files containing the scripts of all data analysis

  Moreover, meta-data of raw RNA-Seq data of B. mori must be shown as in Supplementary Data S8, and the mass spectrometry data must be uploaded in the data repository with DOI, which I showed above.

  <u>Response and action taken</u>: We thank the reviewer for this helpful suggestion and have uploaded all the requested data to figshare currently accessible under

https://figshare.com/s/34742f1cdc7428c0d88c. The data will be made publicly available with its respective doi upon acceptance for publication. We also now refer to the deposited data in the revised manuscript under the data availability chapter. Raw proteome data and transcriptome data were deposited in the commonly used repositories for such data sets. As stated in the data availability part of the manuscript transcriptome data can be found in SRA (Sequencing Read Archive) under accession number PRJNA1096142 (reviewer token https://dataview.ncbi.nlm.nih.gov/object/PRJNA1096142?reviewer=t9hpdhv05mc6oidb8f65aimtu). The mass spectrometry data has been uploaded to PRIDE (PRoteomics IDEntifications database) under the accession number PXD051369 (reviewer token Username: reviewer_pxd051369@ebi.ac.uk, Password: SF54ygw3). They will be made public upon acceptance of the manuscript. Additionally, the respective metadata detailing all timepoints and their corresponding SRA and PRIDE identifiers have been added in Supplementary Data S17 and are referred to in the data availability chapter.

- The other point is that comprehensive workflow of data analysis must be added in main figure to make the readers better understanding the article (as figure 1 of Zheng et al., (2024), URL: https://www.nature.com/articles/s41597-024-03855-3, or figure 1 of Rahuman et al., (2024), URL: https://www.nature.com/articles/s41597-024-03541-4).

Response: We appreciate this valuable suggestion to include a schematic illustrating the comprehensive data analysis workflow.

Action taken: To enhance reader understanding, we have created a figure that visually represents the workflow. Given its detailed nature and role as a reference, we have included it in the supplementary figures as Figure S1 and refer to the figure in the main text and in the methods paragraph. This placement ensures accessibility for readers seeking additional clarity while maintaining the flow of the main text. We believe this approach optimally supports the overall presentation of the manuscript.

- In line 144, the authors described lack of GO annotation in B. mori. I suggested GO enrichment analysis using other annotation data in Bono et al., (2022) (URL: https://doi.org/10.3390/insects13070586).

  Response: We appreciate this valuable suggestion. We believe to have taken a similar approach for the functional annotation of the *Bombyx mori* transcriptome and proteome. In revising the manuscript, we realized that our annotation methodology could be clarified further in the main text, so we have revised it to better highlight our approach. A key difference between our approach and that of Bono et al. (2022) is that we used SonicParanoid with MMSeq2 sequence alignment to predict orthologous relationships, rather than relying on BLAST for sequence similarity. We focused specifically on orthologous relationships between *B. mori* and *D. melanogaster*, the closest model organism with extensive functional information, to infer functional annotations. Additionally, while Bono et al. (2022) provides a useful tool for functional annotation, their dataset did not include GO terms for *B. mori*. To address potential biases associated with inferring functional annotations from orthology alone, we also performed direct Pfam domain predictions based on *B. mori* protein sequences, providing an additional layer of species-specific information. We believe our methodology is well-suited to the functional annotation of *B. mori* genes and, with the clarification added to the text, respectfully suggest retaining our original analyses.

  Action taken: We revised the main text to include the following section:

  "To this end we inferred GO annotations for *B. mori* by utilizing orthologous relationships with *D. melanogaster*. We complemented GO annotations with direct Pfam predictions based on *B. mori* protein sequences to provide species-specific protein family and domain information."

Minor points

1. Accession IDs of genome data of D. melanogaster and B. mori must be added in "Methods" part (lines 744-749).

Response: Done.

2. URLs and references of all data analysis tools must be shown (lines 732-737).

Response: We now added the respective information for all used analysis tools to Supplementary Data S16 and refer to it in the methods part.

Reviewer #2 (Remarks to the Author):

In this manuscript the authors collected transcriptomic and proteomic data along with the development of the silkworm from egg to adult. This is a novel attempt since these data are obtained as pairs and could be of relevant scientific value. However, I have two major concerns for the publication of the manuscript.

1. The title refers to the study as systems biology and indeed it contains some systems biological analyses, but they hardly occupy major portion of this study. I'd rather feel this paper as a bioinformatics study.

Response: We appreciate the reviewer's recognition of the scientific significance of our work and agree that adjusting the title, as suggested, would enhance the manuscript.
Action taken: We changed the title to: "Unraveling developmental gene regulation in holometabolous insects through comparative transcriptomics and proteomics".

2. Reproducibility of the data
Every study that refers to development must be conducted with the extreme cares on the staging of the organism since that is the only way to describe objectively what is under analysis. Detailed description of the embryonic development based on the morphology of Bombyx mori embryo can be found in several literatures and the authors should be aware of it. One can easily describe the embryonic stages of the samples by observing embryos by dissecting small numbers of the eggs if they are well synchronized. Post-embryonic development of insects should be described in concern with the molting cycle since many biological processes are regulated in accordance with the molting cycle which is in vast part controlled by the endocrinological conditions. Therefore, in this context the authors should provide at least on which day of which instar they collected samples, not the days after hatching. Moreover, the larval duration widely varied under the rearing conditions provided, suggesting the rearing condition was suboptimal. Nevertheless, without providing standard results of breeding (e.g. duration of each instar) for the strain used, one cannot evaluate the data in terms of silkworm biology.

Response: We appreciate the reviewer's concern about accurate staging in developmental studies. While we acknowledge that dissection-based staging of embryos would provide more detailed information, our study relied on egg color changes as a non-invasive method to approximate developmental timepoints. This is for example supported by Yamashita and Hasegawa (1983) (https://febs.onlinelibrary.wiley.com/doi/pdf/10.1016/0014-5793(83)80949-1), where natural changes in egg coloration were used to monitor embryonic development in *B. mori* and track RNA and protein synthesis during embryogenesis. For future studies, we acknowledge that incorporating dissection-based staging for a subset of samples would provide valuable validation. However, for the current study, we consider egg coloration to be a phenotypic marker for determining distinct embryonic developmental timepoints.

We also appreciate the reviewer's comments regarding post-embryonic development and its regulation by molting cycles. We agree that molting cycles are critical regulators of many biological processes in insects. However, our study was not designed to investigate gene expression changes specifically associated with molting cycles but rather aimed to capture broader stage-specific transcriptomic and proteomic profiles across larval development. Our sampling strategy included six timepoints during larval development, which were spaced too far apart to resolve fine-scale temporal changes related to molting cycles (e.g., shortly after molting versus later feeding phases). To robustly investigate molting-related processes, future studies will need to sample at much shorter intervals within each instar phase while accounting for variability in molting durations between individuals under different rearing conditions. We also note that *B. mori* has five larval instars compared to *Drosophila melanogaster*, which has three instars with different durations and endocrinological regulation. Given these differences, our dataset was not intended to directly address molting-associated processes but rather provide insights into stage-specific transcriptome-proteome correlations across species.

Action taken: To clarify this limitation of our study design, we have added the following statement to the discussion: "*B. mori* has five larval instars, while *D. melanogaster* has three instars resulting in different developmental durations and endocrinological regulation[100]. For better comparison the collection of silkworm larvae was not synchronized according to the molting cycle, hence gene expression changes associated with molting cannot be compared directly."

3. It looks inevitable to reevaluate all the data, and my suggestion is to concentrate on the earliest two stages which may mark the switching of maternal and zygotic gene expression and protein production.

Response: We thank the reviewer for this insightful comment. Indeed when looking at the correlation heatmap of both transcriptome and proteome samples (Supplementary Fig. S4 and S3, respectively) an obvious shift can be observed between the freshly laid white eggs and the brown eggs which are a few hours old, indicating distinct gene expression in these two developmental timepoints. Taking into consideration that the freshly laid eggs (white eggs) are not yet fertilized (Kawamura, 1978; https://onlinelibrary.wiley.com/doi/abs/10.1002/jmor.1051580106) the detected mRNAs are most probably maternally deposited. To further examine the potential maternal genes we cross-referenced our data with a previously established set of 1,534 maternal genes in *D. melanogaster* (Hooper et al., 2007; https://www.embopress.org/doi/full/10.1038/msb4100112*)*. Indeed, when overlapping transcripts that are significantly more abundant in egg white when compared to egg brown, we found a significant number of orthologs are enriched with Drosophila maternal genes. At the proteome level we did not observe such enrichments, which is expected taking into consideration that maternal genes are primarily deposited as mRNAs rather than proteins (Lorenzo-Orts and Pauli, 2024; https://pmc.ncbi.nlm.nih.gov/articles/PMC11088918/). A GO term enrichment analysis of the transcripts that are significantly more abundant in egg white representing potential *B. mori* maternal transcripts showed DNA metabolism related functions overrepresented, which have been previously reported in maternal genes (Hooper et al., 2007; https://www.embopress.org/doi/full/10.1038/msb4100112). Therefore we agree with the reviewer that our manuscript should also report these results together with a list of potential maternal genes and genes expressed upon the maternal to zygotic switch in *B. mori*.

Action taken: We now added the following paragraph and relevant supplementary tables to the results: "Interestingly, white eggs (Ewhite), the earliest timepoint, clusters separately from the other egg timepoints. This is also evident in the correlation heatmaps of both transcriptome and proteome samples (Supplementary Fig. S4 and S3, respectively). As the fusion of sperm and egg pronuclei occurs within a few hours after oviposition[56], the transcripts and proteins detected in Ewhite are most likely maternally deposited. The observed shift in expression between Ewhite and brown eggs (Ebrown) may reflect the onset of zygotic transcription following fertilization. To further investigate potential maternal genes, we cross-referenced our dataset with 1,534 previously established maternal genes

in *Drosophila melanogaster*[57]. Notably, among the 2,101 transcripts significantly more abundant in Ewhite compared to Ebrown (FDR < 0.05, fold change > 2; Supplementary Data S4), we identified a significant enrichment of *D. melanogaster* maternal genes (p = 0.0009, one-tailed Fisher's exact test). However, at the proteome level, no such enrichment was observed (p = 0.15, one-tailed Fisher's exact test; Supplementary Data S5), which is expected taking into consideration that maternal genes are predominantly deposited as mRNAs rather than proteins[58]. A functional enrichment analysis of transcripts significantly more abundant in Ewhite than in Ebrown, representing potential *B. mori* maternal transcripts, revealed an overrepresentation of DNA metabolism-related functions (Supplementary Data S6). This finding is consistent with previously reported functions of maternal genes[57]. Collectively, these results suggest that the earliest two time points, Ewhite and Ebrown, may capture the transition from maternal to zygotic gene expression."

COMMSBIO-24-3689B Point-by-point responses to the referee's comments (Revision 2)

Reviewer #1 (Remarks to the Author):

The authors well addressed my comments. I deeply appreciate their efforts. I recommend the manuscript is worth the publication.

<u>Response:</u> We thank the reviewer for the positive evaluation of our revision and are pleased that they consider the manuscript suitable for publication.

Reviewer #2 (Remarks to the Author):

Through the revision of the manuscript, authors are well aware of the comments I placed and though I found them not absolutely satisfactory but acceptable. One issue I have to emphasize is the reproducibility of the results. Since the sampling timings are not staged along the developmental process, especially during larval stage, which authors admit, it is not realistic to reproduce the results shown in this paper by other hands. In my opinion, addition of good reasoning to claim the significance of the data shown (even without staged sampling) will avoid spoiling the value of this paper.

<u>Response:</u> We appreciate the reviewer's concern regarding reproducibility and the lack of strict staging according to morphology especially in the larval time points. We fully agree that detailed staging according to morphology can enhance precision and reproducibility in developmental studies. Our experimental setup was designed to identify global and stage-specific patterns, such as transcript-protein correlation and functional enrichments, and to enable comparative insights through alignment with the *Drosophila melanogaster* developmental time course. Importantly, the datasets generated in this study represent the first integrated developmental proteome and transcriptome resource for *B. mori*, offering a valuable foundation for future research in silkworm biology and for comparative analyses with other insect models. Throughout the manuscript, we underscore the significance of our study by

demonstrating the high quality of our omics datasets and highlighting their consistency with findings from other studies across various levels. We believe that the relevance of our research findings has already been adequately emphasized.

Action taken: To nevertheless discuss the limitation of our sampling approach and provide context for readers, we have now added an explanatory paragraph to the discussion section of the manuscript:
"The aim of our study was to capture broad stage-specific transcriptomic and proteomic profiles across the silkworm life cycle using a sampling strategy based on timing rather than morphology throughout development. However, this sampling strategy has limitations with regard to certain research questions. In future studies sampling according to morphology would enable investigating molting-related processes."

Minor points

L54 I assume authors want to mean "80% of the species from this class (if referring Insecta) or subphylum (if referring Hexapoda)".

Response: Done.

L56 Stages of hemimetabolous insect are egg, nymph, adult and nymph is not considered as equivalent to larva, so it is inaccurate to describe it as if it is only lacking pupal stage.

Response and action taken: We agree with this notion and removed this part of the sentence as it has no relevance to the rest of the manuscript.

L58 "Holometabola comprises various insect orders"

Holometabola is insect so using "insect order" for describing it is apparently duplicated expression. In addition, it is not clear what kind of variation the authors

want to mean by including “various”. In my opinion “Holometabola comprises 11 orders,” is appropriate here.

Response and action taken: Agreed and changed the text as suggested.

L67 “D. melanogaster and B. mori, which belong to a monophyletic group”

This is quite confusing since all the insects belong to a monophyletic group, all the holometabolous insects belong to yet another monophyletic group. I suggest including the name of this clade Mecopterida (or Panorpida). Reference 37 is not appropriate here.

Response and action taken: We thank the reviewer for catching this imprecise statement and changed the respective text to use “Mecopterida”, removed the original citation and added a more appropriate one.

L91 “crawling upward to prepare for pupation”

Proper technical term is “wandering”

Response and action taken: Agreed and changed the text to “wandering” as suggested.

L92 “pupa” -> pupae

Response and action taken: Agreed and changed the text as suggested.

L124 To my knowledge, it is not widely known that fibrohexamerin (formerly known as P25) gene is expressed during pupal stage, since it’s believed that the expression of this gene is restricted to the posterior silk gland, and this tissue degenerates during pupal molt. I urge the authors to provide references if this expression timing has already been reported, otherwise please provide insight on why you are detecting this, and which tissue is the provisional expression site. Likewise, cocoonase gene

expression in adults is also not expected. These might be minor points in the context of this study, but quite intriguing results.

Response 1: We thank the reviewer for highlighting the current understanding of P25/fibrohexamerin expression. We have not found any literature analysing P25 expression during the pupal stage. When looking into RNA expression of P25 in our data we detect expression at the first pupal time point P0. Our findings therefore suggest persistence of P25 proteins in the later pupal timepoints. Using our data we cannot clarify the source of this expression. Protein expression is nevertheless decreasing steadily in late pupae further suggesting no active new expression of this protein.

Action taken: We describe this discrepancy and provide an additional Supplementary Figure in the revised manuscript: "The protein P25, a major component of silk fibroin, was found in larvae and pupae. This trend can also be seen in our transcriptome data (Supplemental Fig. S4). So far, P25 expression has only been shown in larvae (Tao et al., 2025; https://www.sciencedirect.com/science/article/pii/S0141813025026662)."

P25 RNA expression in the time-course of our study

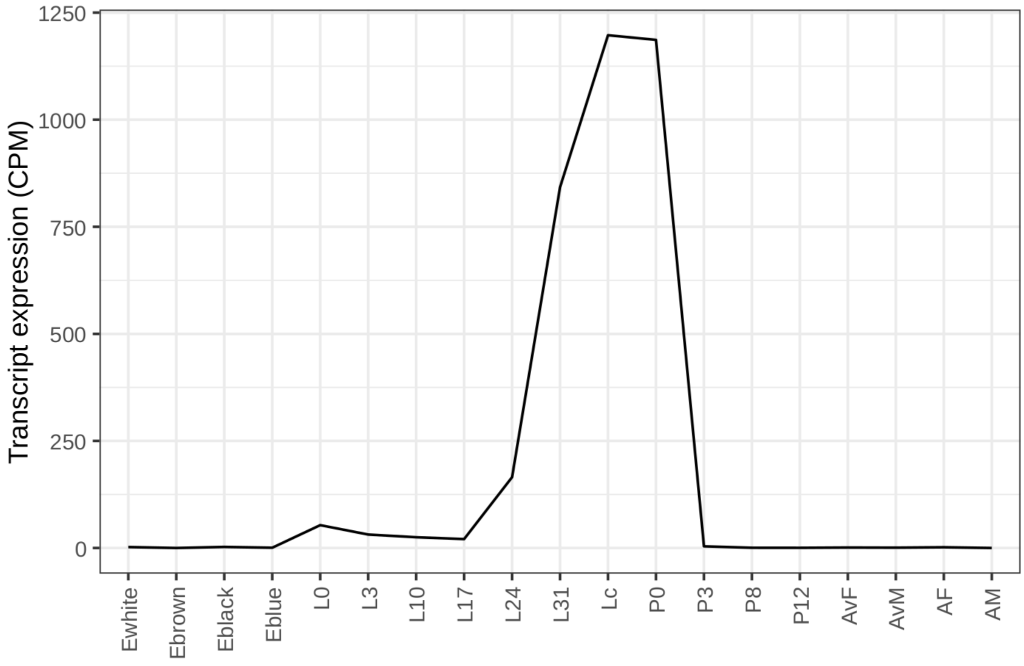

Response 2 and action taken: We thank the reviewer for highlighting this point. Previous studies have shown that cocoonase is specifically expressed during the late pupal stage, peaking just before adult emergence, with transcript levels declining rapidly thereafter (Gai et al., 2020; https://pmc.ncbi.nlm.nih.gov/articles/PMC7544147/). The enzyme is synthesized as an inactive zymogen and stored in the head, where it is later activated to facilitate cocoon digestion during eclosion (Phoeurk et al., 2021; https://www.sciencedirect.com/science/article/pii/S1046592821001029). Our transcriptome data align with these findings (see plot below), showing expression in the last pupal and early adult stages, but not in older adults. The continued detection of cocoonase protein in older adults likely reflects residual protein stores, as the enzyme is no longer actively transcribed at this stage. SilkDB data confirms high expression in the head of newly emerged moths, consistent with its known site of synthesis and storage. Thus, our results are in agreement with the established literature, and the presence of cocoonase protein in old adults is likely due to protein stability rather than ongoing gene expression. In light of this, we would suggest refraining from making any changes to the manuscript regarding this point.

Cocoonase RNA expression in the time-course of our study

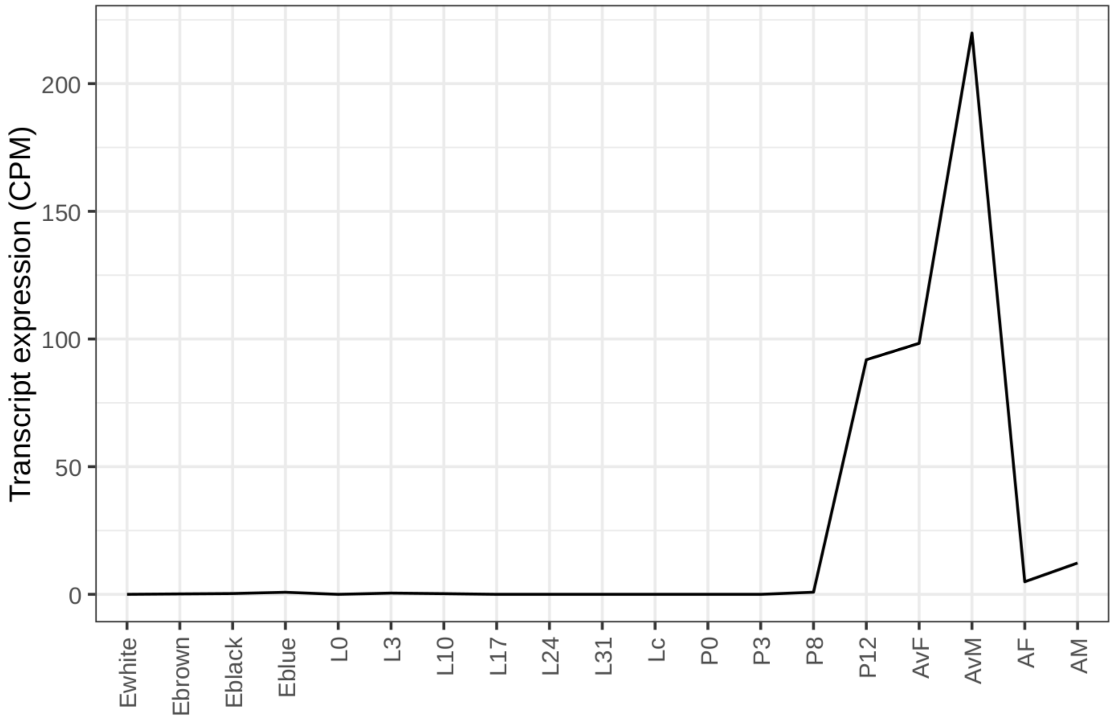

L125 pupation -> pupae

Response and action taken: Agreed and changed the text as suggested.
